# Antiviral Activity and Mucosal Safety of Novel Human-Milk-Inspired Antimicrobial Peptides

**DOI:** 10.3390/pharmaceutics18080938

**Published:** 2026-07-30

**Authors:** Nazita Yousefieh, Louise A. Ouattara, Maimoona Bhutta, Ishita M. Shah, Carolina Herrera, Gustavo F. Doncel

**Affiliations:** 1 CONRAD, Eastern Virginia Medical School, Macon & Joan Brock Virginia Health Sciences, Old Dominion University, Norfolk, VA 23507, USA; yousefn@odu.edu (N.Y.); ouattaal@odu.edu (L.A.O.); bhuttam@odu.edu (M.B.); herrerc@odu.edu (C.H.); 2 Foods for Health Institute, Department of Food Science and Technology, University of California Davis, Davis, CA 95616, USA; ishita.shah@matrubials.com; 3 Matrubials Inc., 630 Pena Drive, Suite 100, Davis, CA 95618, USA

**Keywords:** peptides, HIV-1, HSV-2, female genital tract, broad-spectrum activity, sexually transmitted infections

## Abstract

**Background**: Sexually transmitted infections (STIs) remain a major global health concern, with over one million new curable cases reported daily and limited long-term treatment options. Additionally, there are limited treatment options for women with bacterial vaginosis (BV), a dysbiotic condition predisposing them to STIs. Computationally designed human-milk-inspired antimicrobial peptides (AMPs) are promising candidates for novel antiviral and antibacterial therapies. This study evaluated the cervicovaginal mucosal safety and antiviral activity of three human-milk-inspired AMPs (MAT-006, UCD-MAT-001, and UCD-MAT-002) previously shown to exhibit antimicrobial activity against BV-causing pathogens. **Methods**: AMP cytotoxicity and inflammatory responses were assessed in relevant genital tract cell lines and cervicovaginal tissues in vitro. AMP anti-HIV-1 and anti-HSV-2 activities were characterized in female genital tract cell lines, TZM-bl cells and HEC-1-A cells, respectively, and in time-of-addition (pre-, co-, and post-HIV-1 challenge; and post-HSV-2 challenge) experiments. The impact of seminal plasma on AMPs’ activity was also evaluated. **Results**: At the concentrations tested, all three AMPs showed minimal cytotoxicity and no pro-inflammatory responses in cellular and ectocervical tissue explant models. In both co- and post-exposure models, all three AMPs inhibited HIV-1 and HSV-2 replication with IC_50_ values ranging from 0.78 to 7.72 mg/mL. MAT-006 further showed pre-exposure anti-HIV-1 activity after 6 h (IC_50_ = 1.07 ± 0.515 mg/mL) and 24 h (IC_50_ = 0.69 ± 0.152 mg/mL) of incubation. Seminal plasma did not significantly impair MAT-006 and UCD-MAT-001 antiviral activity under experimental conditions. **Conclusions**: These findings demonstrate the antiviral activity of human-milk-inspired AMPs against HIV-1 and HSV-2 at concentrations that are safe to the cervicovaginal mucosa in vitro, warranting further preclinical evaluation as microbicide candidates with dual antibacterial and antiviral properties.

## 1. Introduction

Every day, more than one million new cases of curable sexually transmitted infections (STIs) are reported worldwide among individuals aged 15 to 49 years, with most of these cases being asymptomatic. According to UNAIDS, approximately 41 million people globally were living with HIV in 2024, and 1.3 million people became newly infected [1]. Women and girls accounted for 45% of all those new infections. Additionally, approximately 520 million new genital herpes (HSV-2 infection) cases were reported [2,3,4].

The vaginal microbiota plays a crucial role in maintaining a healthy physiological environment in the female genital tract (FGT). Bacterial vaginosis (BV) is a disruption of the vaginal ecosystem, often asymptomatic, characterized by a significant shift from a *Lactobacillus*-dominant state to a dysbiotic condition due to an intense proliferation of anaerobic bacteria, including *Gardnerella vaginalis*, *Prevotella* spp., *Mobiluncus* spp., *Mycoplasma hominis*, and *Atopobium vaginalis* [5,6,7]. BV is the most prevalent vaginitis in females of reproductive age, with an occurrence of 20–29% globally [5]. Although BV is not considered to be an STI, this dysbiotic condition is correlated with an increased risk of contracting STIs, such as HIV, chlamydia, and HSV-2 [8,9,10,11,12]. Current treatment options for BV include broad-spectrum antibiotics such as metronidazole (a 500 mg oral dose twice a day for 7 days or 0.75% intravaginal gel applied once a day for 5 days), clindamycin (2% intravaginal gel applied once a day for 7 days) [13], or more recently, secnidazole (2 g single oral dose) [14]. Furthermore, as many as 50% of women with BV experience recurrence within 1 year of treatment [15]. Metronidazole and clindamycin have been shown to lack long-term effectiveness, with recurrence rates as high as 35% within the first three months and 60% within a year following treatment. Additionally, studies have shown in vitro antibiotic drug resistance in BV-associated bacteria [16,17] and in vivo antibiotic drug resistance in women with recurrent and refractory BV [18,19]. The alarming rise in antimicrobial resistance has prompted scientists to actively pursue alternative treatment candidates with long-term effectiveness [20,21].

Despite the availability of oral and injectable antiretrovirals for HIV prevention, antiretroviral resistance is becoming increasingly prevalent [22]; hence, compounds with alternative mechanisms of HIV inhibition are highly desirable. While genital herpes infection does not significantly impact the mortality of otherwise healthy adults, it affects more than one in five adults worldwide [23], and current antivirals are also subject to the development of resistance [24,25]. One promising approach is the development of peptide-based antivirals [26,27,28,29,30,31,32,33,34,35]. In addition to their antiviral activity, several studies have reported AMPs that can treat bacterial infections in humans, potentially replacing antibiotics, especially for the treatment of resistant pathogens [36,37]. The evaluation of innate immune factors in the female genital tract has shown that the vaginal fluids of women with BV are deficient in antimicrobial peptides (AMPs) compared to those of healthy women [38].

Human breast milk is considered the ideal feeding regimen for newborn infants, providing developmental nutrients, shaping their immune system, establishing adequate tolerance to their environment, and developing microbiota. Multiple peptides derived from milk have been described to have physiological and metabolic functions for human health, including antibacterial and antifungal activity [39,40,41,42,43,44]. Multifunctional milk-inspired AMPs are known to limit microbial invasion and replication in host cells, in addition to reducing inflammation and enabling homeostasis [38].

Multiple natural and synthetic peptides have shown antiviral activity via such mechanisms. Bovine lactoferricin has been demonstrated to inhibit HSV-1 and HSV-2 infection by blocking viral entry [26]. Three whey-protein-derived peptides (IAEK, IPAVF, and MHI) exhibited activity against SARS-CoV-2, preventing cell-to-cell fusion [27]. DP178, a synthetic peptide of the HIV-1 Env glycoprotein 41 (gp41) gene, was reported to be a potent inhibitor of virus-mediated cell-to-cell fusion [35]. Several synthetic peptides, including T1249, C34, L’644, E1P47, and enfuvirtide (T20), which has been approved for the treatment of patients unresponsive to other antiretrovirals, have been shown to inhibit HIV fusion to target cells [28,29,30,31,32,33,34].

New antimicrobial therapeutics with broad activity against HIV/HSV and BV pathogens that can simultaneously preserve the healthy lactobacilli-dominant microbiome would represent a major advance in the treatment of BV and contribute to reducing the risk of HIV/HSV transmission.

In a study published in this issue by Shah et al. [45], three computationally designed human-milk-inspired AMPs (UCD-MAT-002, UCD-MAT-001, and MAT-006) were tested for their antibacterial activity against BV. These peptides inhibited the growth of anaerobic bacteria (*Gardnerella vaginalis*, *Atopobium vaginae*, *Mageeibacillus indolicus,* and *Mobiluncus curtusii*) without causing a decrease in *lactobacilli* species viability. Here, we evaluate the antiviral properties of these AMPs against sexually transmissible viruses, HIV-1 and HSV-2, and their mucosal safety in cells and tissues representative of the FGT.

## 2. Materials and Methods

### 2.1. Cell Lines, Ectocervical Tissues, Seminal Plasma, AMPs, and Viral Cultures

Cell culture reagents were purchased from Invitrogen Life Technologies (Waltham, MA, USA) unless otherwise specified. All cell and tissue cultures were maintained at 37 °C in an atmosphere containing 5% CO_2_.

VK2/E6E7 vaginal keratinocyte cells (CRL-2616, ATCC, Manassas, VA, USA) were maintained in keratinocyte serum-free medium supplemented with 50 μg/mL bovine pituitary extract, 0.1 ng/mL epidermal growth factor (EGF), 0.4 mM CaCl_2_, 100 U/mL penicillin (ThermoFisher Scientific, Waltham, MA, USA), and 100 µg/mL streptomycin (ThermoFisher Scientific, Waltham, MA, USA). HEC-1-A human endometrial adenocarcinoma cells (HTB-112, ATCC, Manassas, VA, USA) were cultured in McCoy’s 5A media (ATCC, Manassa, VA, USA) with 5% fetal bovine serum (FBS) (ATCC, Manassa, VA, USA), 100 U/mL penicillin, and 100 µg/mL streptomycin.

TZM-bl cells (HRP-8129, BEI Resources, Manassas, VA, USA) from a luciferase reporter HeLa cell line derivative stably expressing high levels of CD4 and CCR5 were cultured in DMEM media containing 10% FBS, 100 U/mL penicillin, and 100 µg/mL streptomycin [46].

Ectocervical tissues were obtained from healthy, HIV-1 negative women who had given their consent and who were undergoing routine elective surgeries for benign indications as part of an Eastern Virginia Medical School (EVMS) IRB-approved protocol (09-09-FB-0175). Ectocervical specimens were obtained from five donors undergoing hysterectomies. Collected ectocervical tissues were immediately transferred to RPMI 1640 media containing 10% FBS, 100 U/mL penicillin, and 100 µg/mL streptomycin (cRPMI). Ectocervical tissues were cut into pieces that were 3 mm in diameter and 2–4 mm thick, referred to as ectocervical explants (ECEs), and cultured overnight in cRPMI medium. The culture medium was replenished every 3–4 days by replacing half of its volume with fresh medium.

Human semen was obtained from healthy normozoospermic men enrolled in an Eastern Virginia Medical School Institutional Review Board (IRB)-approved semen donation program (13-02-FB-0031). Semen was left at room temperature for about 1 h to allow liquefaction to occur. Samples were then centrifuged at 4000 r.p.m. for 20 min at room temperature. Seminal plasma (SP) was collected, aliquoted, and stored at −80 °C. Pooled SP from 5 different donors was used in the experiments.

The AMPs, cationic oligopeptides manufactured by Genscript, Piscataway, NJ, USA at >98% purity, were provided by Matrubials in lyophilized powder form and were stored at −20 °C until used. As described in Shah et al. [45], UCD-MAT-001 was designed as a 12-amino-acid peptide encoded within human β-casein, with four amino acid substitutions compared to the parent sequence and net charge at pH 7 of +5; MAT-006 is a 10-amino-acid peptide encoded within human lactase, with 1 amino acid substitution compared to the parent sequence and net charge at pH 7 of +4; and UCD-MAT-002 is a 10-amino-acid peptide encoded within human α-S1 casein, with 2 amino acid substitutions compared to the parent sequence and net charge at pH 7 of +4.

HIV-1_BaL_, an R5-tropic strain, was a generous gift from Dr. Susana Asin (V.A. Medical Center—Dartmouth Medical School, Lebanon, NH, USA). Viral stocks were generated in PBMCs [47] and titrated in TZM-bl cells according to Kaerber formula [48].

HSV-2 was purchased from The American Type Culture Collection (ATCC, Manassa, VA, USA). Viral stocks were prepared by infecting Vero cells (ATCC, Manassa, VA, USA) and collecting the supernatant after cytopathic effect was observed in the majority of the cells. The supernatant was centrifuged, aliquoted, and stored in −80 °C freezer. HSV-2 stock titer was determined by plaque assay [49]. Briefly, following 2 h adsorption of various virus dilutions onto Vero cell monolayers in a 24-well plate, medium was removed, and cells were overlaid with 1.5% carboxymethylcellulose (Sigma-Aldrich, St. Louise, MO, USA) and incubated for 48 h at 37 °C with 5% CO_2_. After incubation, cells were fixed with 4% paraformaldehyde (Electron Microscopy Sciences, Hatfield, PA, USA) and stained with crystal violet (Sigma-Aldrich, St. Louise, MO, USA). Plaques were counted, and viral titer was calculated as plaque forming units (pfu)/mL.

### 2.2. Cytotoxicity Assays

The impact of milk AMPs on cell viability was assessed using CellTiter 96^®^ Aqueous Non-Radioactive Cell Proliferation [3-(4,5-dimethylthiazol-2-yl)-5-(3-carboxymethoxyphenyl)-2-(4-sulfophenyl)-2H-tetrazolium (MTS)] Assay (Promega, Madison, WI, USA), as per the manufacturer’s instructions. Briefly, VK2/E6E7 and HEC-1-A cells were plated in 96-well plates and treated with serial dilutions of AMPs for 24 h at 37 °C prior to conducting MTS assay. Cytotoxicity in TZM-bl cells was assessed following 48 h exposure to AMPs to mimic experimental setup during evaluation of anti-HIV-1 activity of the AMPs. Untreated cells served as negative control, and cells treated with 0.05 mg/mL nonoxynol-9 (N-9) (generously donated by OrthoMcNeil Corporation) served as positive control [50].

ECEs were treated for 18 h with serially diluted AMPs. The following day, tissue explants were placed in a 1 mg/mL solution of MTT (3-[4,5-dimethylthiazol-2-yl]-2,5 diphenyl tetrazolium bromide) (Sigma-Aldrich, St. Louise, MO, USA) in media for 2 h. ECEs were then placed in 500 μL of methanol overnight, and absorbance was read at 590 nm. ECEs were dried and weighed, and viability was calculated based on absorbance per mg of tissue compared to untreated control [51].

### 2.3. Immune Response to AMPs in Vaginal Cells

To determine whether the human-milk-inspired AMPs induced any pro-inflammatory responses, HEC-1-A cells and ECEs were incubated in duplicate with serial dilutions of UCD-MAT-002, UCD-MAT-001, and MAT-006 at non-cytotoxic concentrations (based on cytotoxicity assays described above) for 24 h and 48 h. Culture supernatants were then collected for measurement of 10 inflammation-relevant cytokines/chemokines (interferon-γ (IFN-γ), interleukin-1β (IL-1β), IL-2, IL-4, IL-6, IL-8, IL-10, IL-12p70, IL-13, and tumor necrosis factor-α (TNF-α)) via Meso Scale Discovery (MSD) technology (V-Plex Human Proinflammatory Panel 1 Kit, Rockville, MD, USA) following manufacturer’s instructions.

Evaluation of early immune responses to AMPs at the transcriptomic level [52] was performed in VK2/E6E7 cells following incubation in duplicate with UCD-MAT-002, UCD-MAT-001, and MAT-006 at non-cytotoxic concentrations for 6 h [49]. Hydroxyethyl cellulose (HEC) (Sigma Aldrich, St. Louise, MD, USA) at 1 mg/mL was used as negative control, while tumor necrosis factor-α (TNF-α) [53,54] and imiquimod (IMQ), a toll-like receptor 7 agonist [9,55], were used as positive controls in these experiments. At the end of incubation, total RNA was extracted from treated cells using Trizol (Invitrogen Life Technologies, Waltham, Ma, USA) and purified using RNeasy mini kit columns from Qiagen Sciences (Qiagen, Germantown, MD, USA) according to manufacturer’s instructions. Quantitative real-time PCR (qRT-PCR) (Roche LightCycler carousel-based system using 4.05 software (Roche, Indianapolis, IN, USA) was performed in a 20 μL reaction volume containing 1 μL cDNA using LightCycler FastStart DNA Master SYBR Green I (Roche, Indianapolis, IN, USA) according to manufacturer’s instructions. Expression levels of selected markers for evaluation of mucosal antiviral candidates, apolipoprotein B mRNA editing catalytic polypeptide-like (APOBEC) [56], C-C chemokine ligand 20 (CCL20) [57,58], claudin-1 (CLDN1) [59], C-X-C chemokine ligand 2 (CXCL2) [60], and interleukin-6 (IL-6) [61] were measured as previously described [52].

### 2.4. Anti-HIV-1 Activity and Effect of Seminal Plasma

TZM-bl cells were plated 24 h prior to treatment [46]. AMPs were tested in serial dilutions starting at concentrations shown not to affect viability of TZM-bl cells in the cytotoxicity assay. For a co-exposure setting, TZM-bl cells were exposed in triplicate to different concentrations of AMPs with or without HIV-1_BaL_ (5 × 10^3^ TCID_50_ per well). Under pre-exposure setting, TZM-bl cells were treated in triplicate with multiple concentrations of AMPs for 1, 6, or 24 h, washed, and then exposed to HIV-1_BaL_. For a post-exposure setting, TZM-bl cells were treated with AMPs 2 or 8 h post-challenge with HIV-1_BaL_. Impact of SP was assessed with pooled SP diluted to 10%. Infection levels were measured after 48 h with Bright-Glo Luciferase Assay System (Promega, Madison, WI, USA) following manufacturer’s instructions. Briefly, cells were lysed with 100 µL of Glo Lysis buffer, equal volumes of lysates along with Bright-Glo assay reagent were transferred into a 96-well black microtiter plate, and relative luminescence units (RLUs) were measured. Infection was normalized relative to RLUs obtained for cells exposed to virus in the absence of AMPs (100% infectivity) and to cells not exposed to virus (0% infectivity). As an internal control of inhibitory activity, TZM-bl cells were treated with 100 μg/mL of tenofovir (TFV), an FDAapproved reverse transcriptase inhibitor.

### 2.5. Anti-HSV-2 Activity and Effect of Seminal Plasma

HEC-1A cells were cultured for 24 h prior to exposure to 0.001 MOI of HSV-2 for 1 h [62]. The inoculum was then removed, and cells were incubated with serial dilutions of AMPs (starting at the highest non-cytotoxic concentration) in the presence or absence of 10% pooled SP for 3 days. Untreated control cells were challenged with HSV-2 and then cultured in HEC-1-A cell culture media with no AMPs. HSV-2 viral DNA levels were measured in culture supernatants collected at day 3 by qRT-PCR performed with QuantStudio™ 3 Real-Time PCR Systems (Thermo Fisher Scientific, Waltham, MA, USA) using TaqMan Gene Expression Master Mix (Thermo Fisher Scientific, Waltham, MA, USA) according to manufacturer’s protocol. HSV-2 DNA template (Integrated DNA Technology, SanDiego, CA, USA) was used to create a standard curve to calculate the copy number of HSV-2 DNA in each sample (Table 1). Infection was normalized relative to HSV-2 DNA copy number obtained for cells exposed to virus in the absence of AMPs (100% infectivity) and to cells not exposed to virus (0% infectivity). As an internal control of inhibitory activity, HEC-1-A cells were treated with 1000 μg/mL of TFV [63].

### 2.6. Statistical Analysis

Cytokine/chemokine concentrations, and 50 or 90 percent inhibitory concentration (IC_50_, IC_90_) values were calculated from sigmoid curve fits (Prism, GraphPad). All data presented fulfill the criterion of R2 > 0.7. Ratios between analyte concentrations in cultures treated with AMPs and analyte levels from untreated control cultures were established. Ordinary one-way ANOVA with Tukey’s multiple comparisons tests was used for comparison between AMPs and to test the effect of biological fluids across various drug concentrations. Treatment conditions were considered significant at *p* < 0.05. Differentially abundant cytokine/chemokines were analyzed using Ingenuity Pathway Analysis software (Qiagen, Hilden, Germany) to determine the biological processes affected by each AMP. The pathways with a minimum of two associated analytes at *p* < 0.05 were considered to be enriched.

## 3. Results

### 3.1. Viability and Mucosal Inflammatory Responses in Cell- and Tissue-Based Models

**Figure 1:**
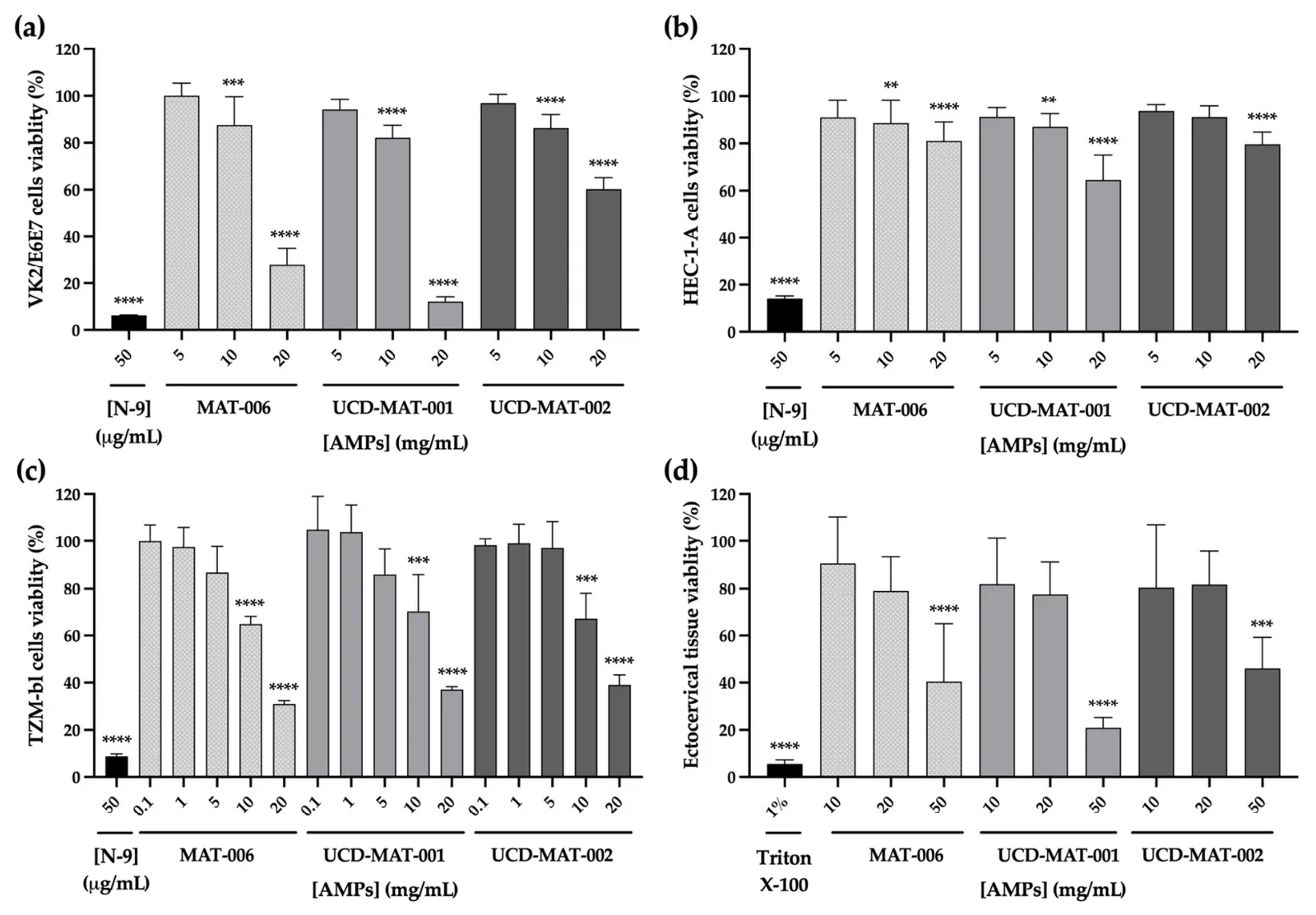
In vitro and ex vivo cytotoxicity of milk AMPs. Vaginal VK2/E6E7 cells (**a**), endometrial HEC-1-A cells (**b**), cervical TZM-bl cells (**c**), and ectocervical tissue explants (**d**) were exposed to serial dilutions of UCD-MAT-002, UCD-MAT-001, or MAT-006. N-9 and Triton X-100 were used as negative controls of viability in cell lines and tissue explants, respectively. The percent viability was determined by MTS assay for cells and by MTT assay for explants and was normalized relative to untreated cells or tissue explants. Data shown are mean (standard deviation) from at least two independent experiments performed in triplicate. Statistical analyses were conducted using ordinary one-way ANOVA with Tukey’s multiple comparison tests. ** *p* < 0.01, *** *p* < 0.001, **** *p* < 0.0001.

We first assessed the potential cytotoxic impact of each AMP in in vitro cellular models representative of the human FGT. Vaginal epithelial VK2/E6E7 cell viability was not significantly affected by any of the three AMPs following 24 h incubation at concentrations ≤5 mg/mL (Figure 1a). However, viability decreased by 14% at 10 mg/mL and 40% at 20 mg/mL when incubated with UCD-MAT-002 (*p* < 0.0001). For UCD-MAT-001, cell viability decreased by 18% at 10 mg/mL and 88% at 20 mg/mL (*p* < 0.0001). Treatment with MAT-006 resulted in a 13% decrease in cell viability at 10 mg/mL (*p* = 0.0004) and a 72% decrease at 20 mg/mL (*p* < 0.0001), compared to untreated cells. The viability of endometrial HEC-1-A cells decreased in a dose-dependent manner following treatment with UCD-MAT-002, UCD-MAT-001, or MAT-006 (Figure 1b). Cell viability significantly decreased with 20 mg/mL of UCD-MAT-002, UCD-MAT-001, or MAT-006 (*p* < 0.0001). UCD-MAT-001 or MAT-006 at 10 mg/mL still caused significant cytotoxicity (*p* = 0.0024 and 0.0095, respectively), while the same concentration of UCD-MAT-002 was not cytotoxic. Treatment with 5 mg/mL of any of the three AMPs did not affect cell viability. AMP concentrations ≤5 mg/mL were not toxic to cervical epithelial TZM-bl cells after 48 h of exposure (≥85% of cell viability observed, Figure 1c).

In an ex vivo ectocervical tissue model, no statistically significant changes in the viability of ECEs incubated overnight with 5, 10, or 20 mg/mL of UCD-MAT-002, UCD-MAT-001, or MAT-006 were observed when compared to untreated explants. However, significant decreases in ectocervical explant viability were observed with 50 mg/mL of all three AMPs tested (Figure 1d). Compared to untreated explants, exposure to 50 mg/mL of MAT-006 resulted in a 60% decrease in viability (*p* < 0.0001), an 80% decrease with UCD-MAT-001 (*p* < 0.0001), and a 55% decrease with UCD-MAT-002 (*p* = 0.0002).

**Figure 2:**
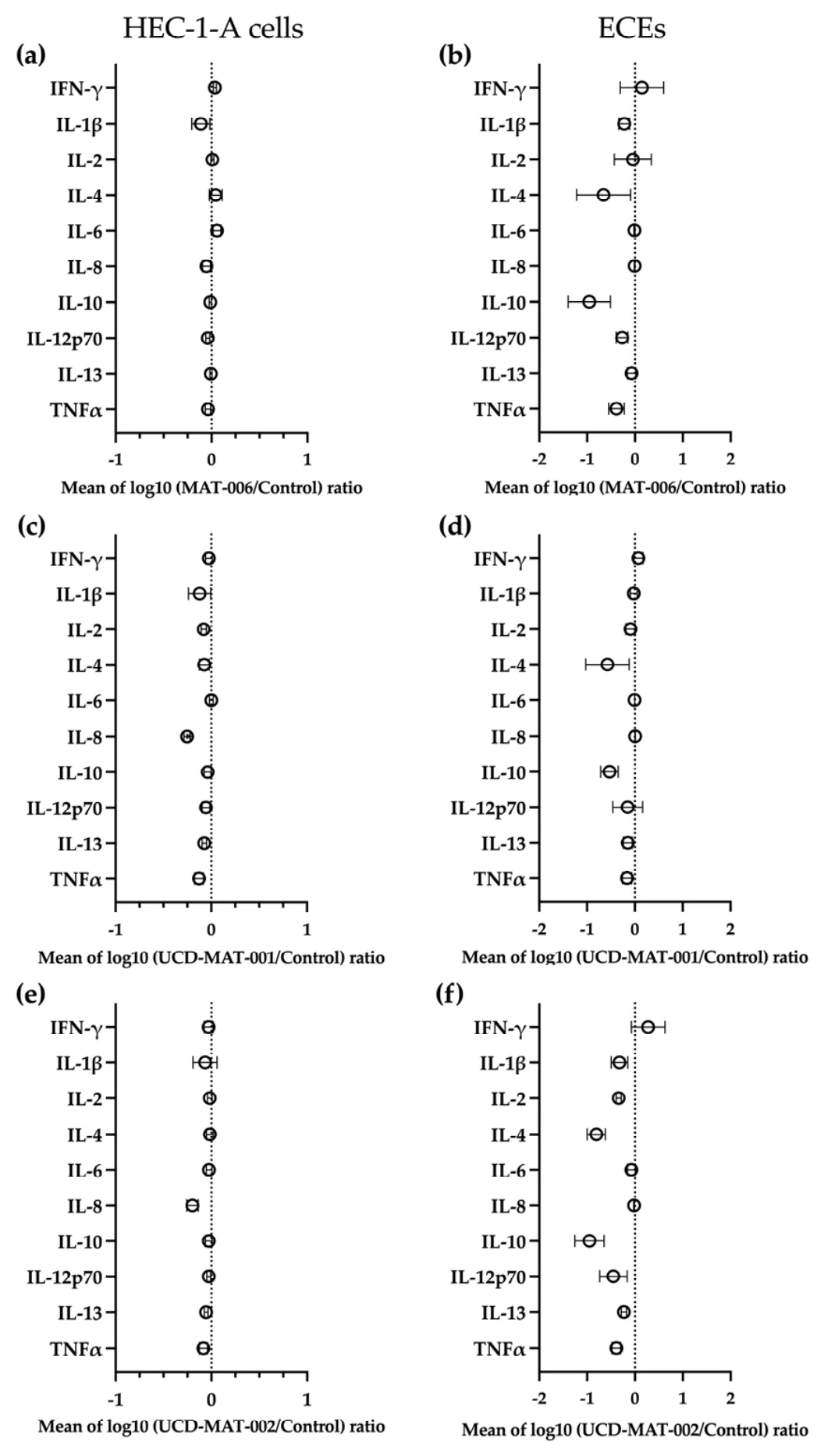
Responses to in vitro and ex vivo exposure to human-milk-inspired AMPs. Endometrial HEC-1-A cells (**a**,**c**,**e**) and ectocervical explants (ECEs) (**b**,**d**,**f**) were treated with 10 mg/mL of MAT-006 (**a**,**b**), UCD-MAT-001 (**c**,**d**), or UCD-MAT-002 (**e**,**f**) for 24 h. Data shown are mean log concentrations of treatments vs. control ratios from at least 2 independent experiments performed in duplicate.

To further assess the FGT responses to AMPs, we measured the levels of inflammation-related cytokines/chemokines secreted in culture supernatants of HEC-1-A cells and ECEs. In both models, after 24 h exposure to AMPs at 10 mg/mL, a high concentration showing minimum cytotoxicity, no inflammatory responses were observed, with a down-modulation of pro-inflammatory cytokine/chemokine secretion (Figure 2). In HEC-1-A cells, UCD-MAT-001 induced a significant decrease in IL-2 (*p* = 0.0032) and TNF-α (*p* < 0.0001) secretions, and levels of IL-8 in culture supernatants were significantly reduced after dosing with UCD-MAT-001 and UCD-MAT-002 (*p* = 0.0121 for UCD-MAT-001, and *p* = 0.0024 for UCD-MAT-002). In ECEs, despite all three AMPs inducing a decreased secretion of adaptive IL-4 (*p* = 0.0115 for MAT-006, *p* = 0.0242 for UCD-MAT-001, and *p* = 0.0111 for UCD-MAT-002) and anti-inflammatory IL-10 (*p* = 0.0110 for MAT-006, *p* = 0.3231 for UCD-MAT-001, and *p* = 0.0114 for UCD-MAT-002), no increase in pro-inflammatory cytokines/chemokines was observed. Similar responses were observed in both HEC-1-A cells and ECEs after exposure to lower concentrations (5 and 1 mg/mL) of AMPs for 24 h (Supplementary Figures S1 and S2). When the time of exposure was extended to 48 h, no inflammatory responses were induced by the AMPs in HEC-1-A cells, while a consistent decrease in IL-8 secretion level was observed with UCD-MAT-001 and UCD-MAT-002 at all concentrations tested (10, 5, and 1 mg/mL) (Supplementary Figure S3). Longer exposure of ECEs to MAT-006 and UCD-MAT-001 did not induce an increased secretion of pro-inflammatory cytokines/chemokines compared to the untreated control ECEs at any of the AMP concentrations tested. With exposure to UCD-MAT-002, a significant increased secretion of adaptive cytokine IFN-γ was only observed in ECEs treated with 10 mg/mL of AMP (Supplementary Figure S4).

Functional pathway analysis of the anti-inflammatory profile induced in HEC-1-A cells after 24 h exposure to AMPs at 10 mg/mL showed down-regulated pathways linked to immune activation and associated with nodes around TNF, IL-2, IL-33, and toll-like receptor 2 (TLR2) for UCD-MAT-001 and with nodes around TNF, IL-1α, IL-1β, IL-2, IL-3, IL-15, IL-33, and TLR3 for UCD-MAT-002 (Supplementary Figures S5 and S6). For MAT-006, no clear down-regulated gene network could be identified in HEC-1-A cells. In ECEs, the anti-inflammatory responses induced by MAT-006 and UCD-MAT-002 were associated with the same functional pathways and nodes around TNF, IL-1α, IL-2, IL-3, IL-6, and IL-33 (Supplementary Figures S7a,c and S8a,c). No clear nodes were obtained via the functional pathway analysis for UCD-MAT-001 in ECEs, but rather genes associated with the down-regulation of T and B cell activation and proliferation (Supplementary Figures S7b and S8b).

**Figure 3:**
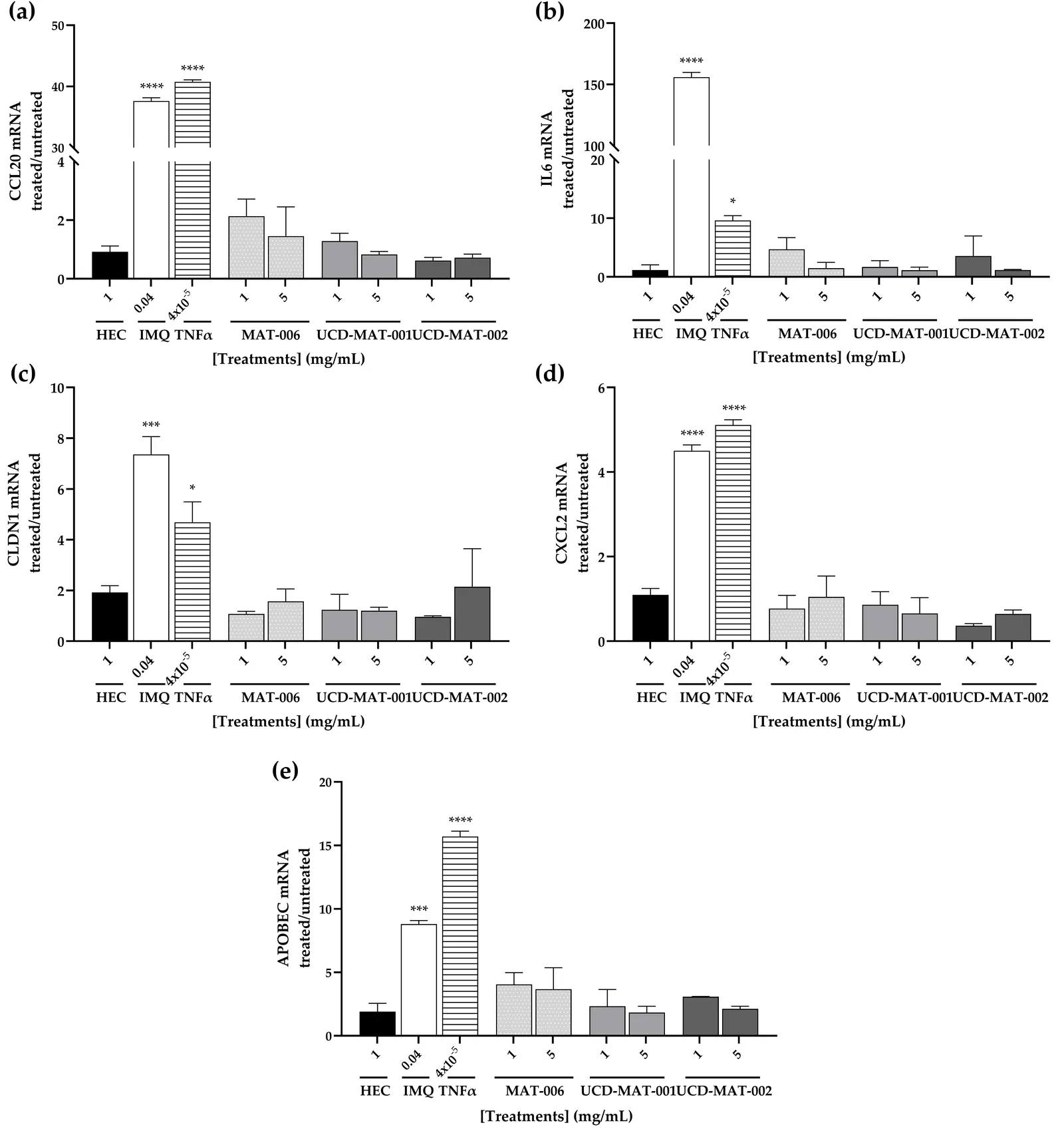
In vitro pro-inflammatory transcriptomic responses to AMPs. Changes in mRNA levels of proinflammatory genes, CCL20 (**a**), IL-6 (**b**), CLDN1 (**c**), CXCL2 (**d**), and APOBEC (**e**), in vaginal VK2/E6E7 cells treated with serial dilutions of MAT-006, UCD-MAT-001, or UCD-MAT-002. 1 mg/mL of hydroxyethyl cellulose (HEC) was used as negative control of inflammation, and 40 µg/mL of Imiquimod (IMQ) and 40 ng/mL of TNF-α were used as positive controls of inflammatory responses. Data shown are means (standard deviation) from at least 2 independent experiments performed in triplicate. Statistical analyses were conducted using ordinary one-way ANOVA with Tukey’s multiple comparison tests. * *p* < 0.05, *** *p* < 0.001, **** *p* < 0.0001.

We previously characterized genes linked to proinflammatory responses in VK2/E6E7 cells treated with microbicides to facilitate the initial screening of candidates before clinical trials [52]. Here, we measured the expression of these biomarkers in VK2/E6E7 cells following incubation with human-milk-inspired AMPs and compared it to changes induced by proinflammatory molecules (TNF-α and IMQ) (Figure 3a–e). Treatment of VK2/E6E7 cells with 40 µg/mL of IMQ or 40 ng/mL of TNF-α significantly elevated the mRNA levels of APOBEC (*p* = 0.0001 with IMQ, *p* < 0.0001 with TNF-α), CCL20 (*p* < 0.0001), CLDN1 (*p* = 0.0001 with IMQ, *p* = 0.0150 with TNF-α), CXCL2 (*p* < 0.0001), and IL-6 (*p* < 0.0001 with IMQ, *p* = 0.0106 with TNF-α). However, there was no significant increase in the mRNA levels of these markers in VK2/E6E7 cells treated with MAT-006, UCD-MAT-001, or UCD-MAT-002 at 1 and 5 mg/mL, compared to untreated cells.

### 3.2. In Vitro Anti-HIV Activity of Human-Milk-Inspired AMPs

**Figure 4:**
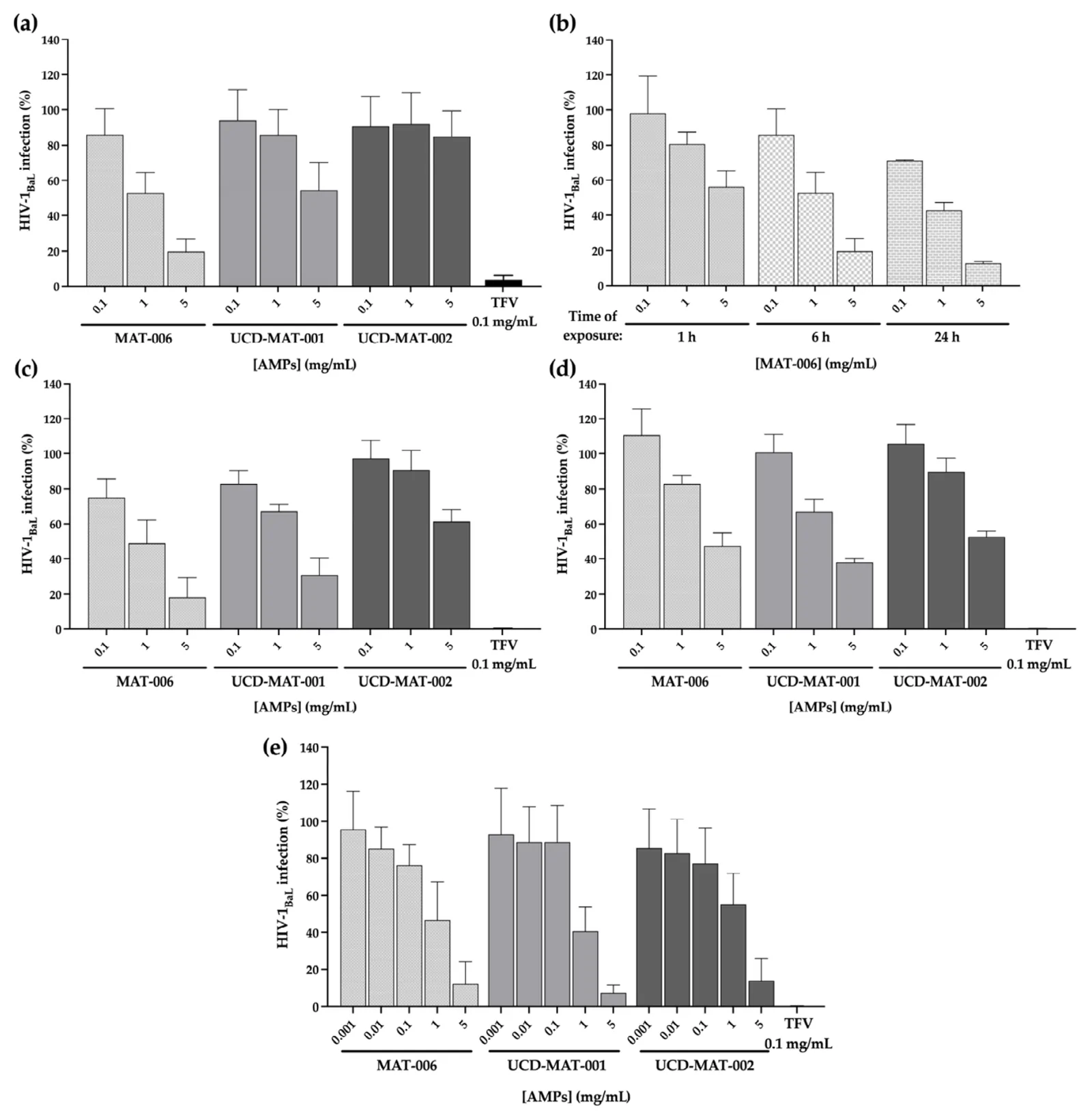
HIV-1_BaL_ infection of TZM-bl cells exposed to AMPs. Cervical TZM-bl cells were treated with serial dilutions of UCD-MAT-002, UCD-MAT-001, or MAT-006 AMPs at different time points relative to viral challenge. Cells were exposed (**a**) for 6 h to milk AMPs, or (**b**) for 1, 6, and 24 h to MAT-006 prior to viral challenge (pre-exposure). Alternatively, cells were challenged with HIV-1_BaL_ and exposed to AMPs either (**c**) 2 h or (**d**) 8 h after challenge (post-exposure); or (**e**) at the same time as viral challenge (co-exposure). Percentage of infection was normalized relative to the relative light units (RLUs) obtained for cells not exposed to virus (0% infectivity) and for cells infected with virus in the absence of AMPs (100% infectivity). Tenofovir (TFV) was included as a positive control of inhibition. Data shown are mean (standard deviation) from at least 3 independent experiments performed in triplicate.

Having determined non-cytotoxic concentrations for each AMP in TZM-bl cells (Figure 1c), we assessed their anti-HIV-1 activity in different exposure models, including the treatment of cells under pre-, co-, and post-viral exposure conditions. When exposing TZM-bl cells to AMPs for 6 h before challenge with HIV-1_BaL_ (pre-exposure dosing), a dose–response curve was observed for MAT-006 within the range of concentrations tested, with an IC_50_ of 1.07 ± 0.515 mg/mL (Figure 4a, Table 2). Only partial or no protection was observed with 5 mg/mL of UCD-MAT-001 (45.5% inhibition, *p* < 0.05) and UCD-MAT-002, respectively. To further characterize the anti-HIV-1 activity of MAT-006 in a pre-exposure dosing setting, we performed a time course evaluation of inhibitory potency, exposing cells to this AMP for 1, 6, and 24 h prior to viral challenge (Figure 4b). Compared to the antiviral potency observed with the pre-exposure of cells to MAT-006 for 6 h, shortening this time to 1 h resulted in a reduction in inhibitory activity (with an estimated IC_50_ = 7.79 ± 5.194 mg/mL), while a slightly increased potency (IC_50_ = 0.69 ± 0.152 mg/mL) was observed with longer pre-exposure dosing times (24 h) (Table 2).

When exposing cells to the milk-inspired AMPs 2 h post-HIV-1_BaL_ challenge, dose–response curves were observed for all three AMPs with the same order of potency observed in the pre-exposure setting reflected by increasing IC_50_ values from MAT-006 to UCD-MAT-001 and UCD-MAT-002 (Figure 4c, Table 2). However, increasing the time between viral challenge and post-exposure dosing resulted in a decrease in inhibitory potency for all three AMPs (Figure 4d, Table 2). In a co-exposure setting, all three AMPs showed inhibitory activity against HIV-1_BaL_ in TZM-bl cells with similar IC_50_ values for all three AMPs (Figure 4e, Table 2).

### 3.3. In Vitro Anti-HSV-2 Activity of Human-Milk-Inspired AMPs

**Figure 5:**
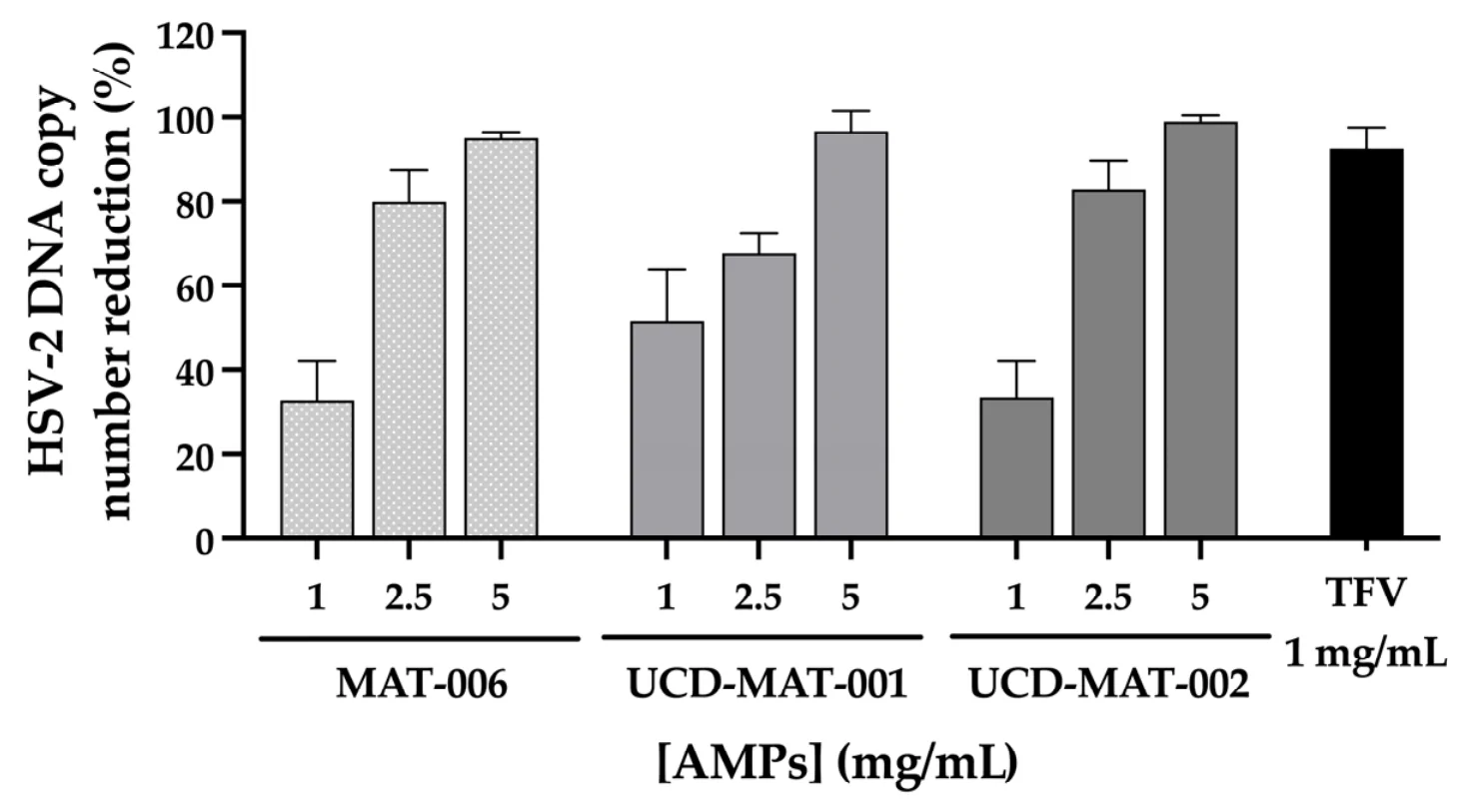
Anti-HSV-2 potency of AMPs in HEC-1-A cells. Cells were treated with UCD-MAT-002, UCD-MAT-001, and MAT-006 post-challenge with HSV-2. Culture supernatants were collected at day 3, and HSV-2 DNA was quantified by qRT-PCR. The percentage of reduction in DNA copy number was normalized to HSV-2 DNA copy number obtained from cells challenged with virus in the absence of AMPs. Percentage of DNA copy number reduction was normalized relative to the DNA copy number obtained for cells not exposed to virus (equivalent to 100% reduction) and for cells infected with virus in the absence of AMPs (0% reduction). Tenofovir (TFV) was included as a positive control of reduction in HSV-2 DNA copy number. Data shown are means (standard deviation) from at least 3 independent experiments performed in triplicate.

To further characterize the antiviral activity of these AMPs, we determined their potency against another highly prevalent viral STI, HSV-2 infection, in an HEC-1-A cell model [64]. Non-cytotoxic AMP concentrations of ≤5 mg/mL (Figure 1b) were used for this study. Treatment of cells with 5 mg/mL of MAT-006, UCD-MAT-001, or UCD-MAT-002 reduced the HSV-2 copy number in HEC-1-A cell culture supernatants by 99% (*p* < 0.0001). Dose–responses were observed for the three AMPs, allowing for the calculation of IC_50_ and IC_90_ values (Figure 5, Table 3). UCD-MAT-001 showed the lowest IC_50_ and IC_90_ values.

### 3.4. Effect of Seminal Plasma on the Antiviral Activity of MAT-006 and UCD-MAT-001

**Figure 6:**
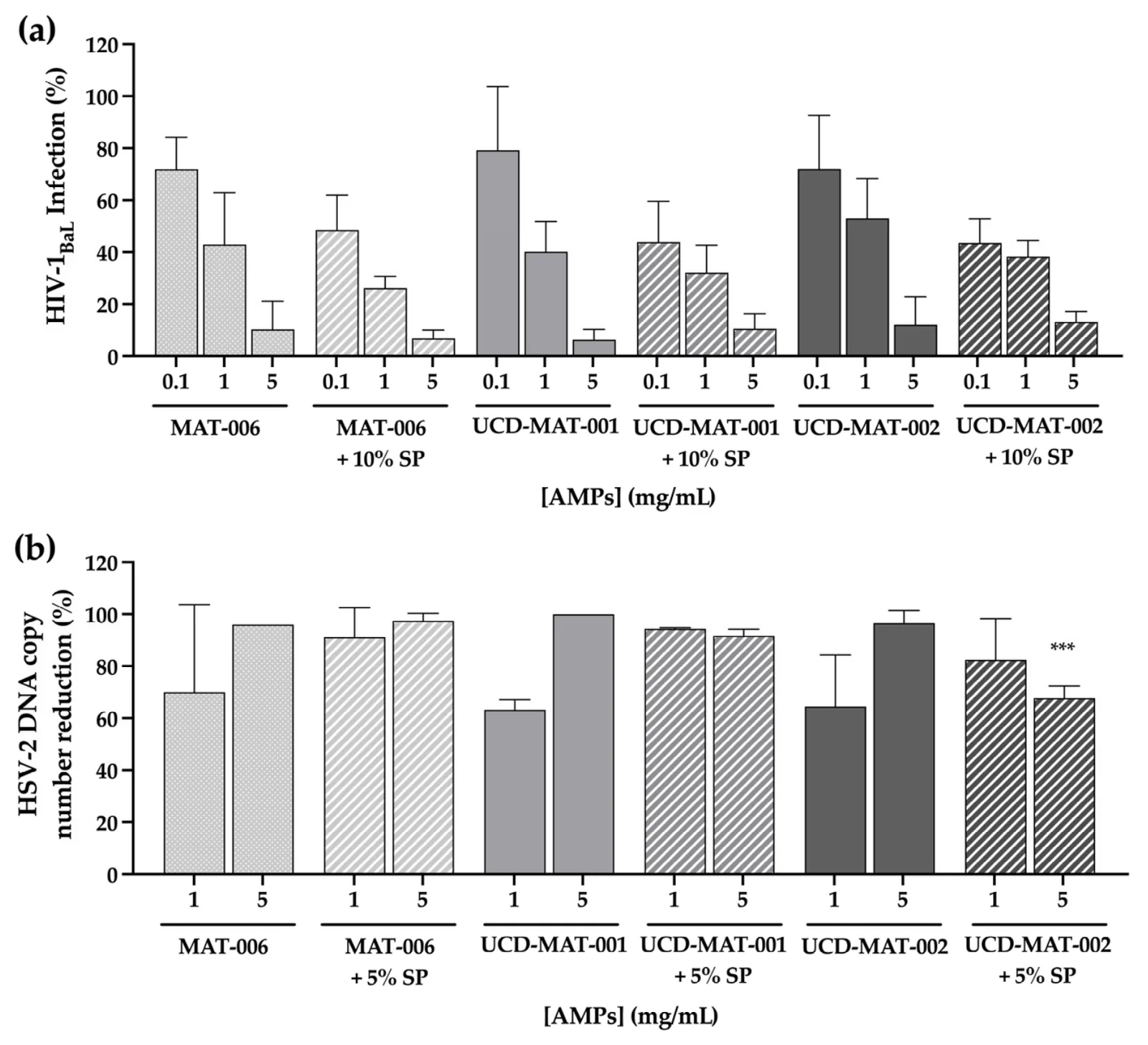
In vitro antiviral activity of milk AMPs in presence or absence of seminal plasma. (**a**) Cervical TZM-bl cells were infected with HIV-1_BaL_ in the presence of the AMPs and +/- 10% seminal plasma (SP). Percentage of infection was normalized against the relative light units (RLUs) obtained for cells not exposed to virus (0% infectivity) and for cells infected with virus in the absence of AMPs (100% infectivity) for assays in the absence or presence of SP. (**b**) Endometrial HEC-1-A cells were infected with HSV-2. Then, AMPs were added +/- 10% SP for 3 days. Percentage of DNA copy number reduction was normalized relative to the DNA copy number obtained for cells not exposed to virus (100% reduction) and for cells infected with virus in the absence of AMPs (0% reduction) for assays in the absence or presence of SP. Data shown are means (standard deviation) from at least 2 independent experiments per cell type performed in triplicate. Statistical analyses were conducted for effect of SP on percentage of reduction at the highest concentration of AMP tested (5 mg/mL) using ordinary one-way ANOVA with Tukey’s multiple comparisons tests. *** *p* < 0.001.

To confirm efficacy of the AMPs in the context of sexual intercourse, antiviral activity was reassessed in the presence of pooled SP. The in vitro HIV-1_BaL_ inhibition level of the AMPs was not significantly affected by the presence of SP in TZM-bl cell cultures (with *p* = 0.678 for MAT-006, *p* = 0.232 for UCD-MAT-001, and *p* = 0.895 for MAT-UCD-002 at the highest concentration tested, 5 mg/mL) (Figure 6a). When SP was added to HEC-1-A cell cultures, the anti-HSV-2 activity of MAT-006 and UCD-MAT-001 was not significantly modulated (with *p* = 0.272 for MAT-006 and *p* = 0.075 for UCD-MAT-001 at the highest concentration tested, 5 mg/mL); however, in the presence of SP, UCD-MAT-002 only reached 67.69 ± 4.712% inhibition at 5 mg/mL, compared to 97.80 ± 3.208% in the absence of SP (*p* = 0.0007) (Figure 6b). The decrease in percent inhibition was not due to increased HSV-2 infection of HEC-1-A cells in the presence of SP compared to the HSV-2 DNA copy numbers measured in the absence of SP.

## 4. Discussion

The novel human-milk-inspired AMPs, MAT-006, UCD-MAT-001, and UCD-MAT-002, identified by the UC Davis Milk Bioactives Program and Matrubials Inc. (a spin-out company from UC Davis), have demonstrated antibacterial activity against BV-associated pathogens [45]. Here, we extended their antimicrobial characterization by evaluating the AMPs’ activities against two clinically prevalent viral STIs, HIV-1 and HSV-2. We tested concentrations in the range of 0.1 to 20 mg/mL, which is the range of products marketed to treat BV, the most common of which contains 7.5 mg/mL of active pharmaceutical ingredient [65]. We showed that these AMPs were not cytotoxic at or below 5 mg/mL and did not induce pro-inflammatory mediators in in vitro and ex vivo cell- and tissue-based models of the cervicovaginal tract, while exhibiting in vitro inhibitory activity against HIV-1 and HSV-2, providing a basis for the further development of these AMPs as a new alternative multipurpose strategy for the topical prevention and treatment of BV and the prevention of HSV-2 and HIV-1 acquisition.

During the preclinical screening of microbicide candidates, an evaluation of the potential inflammatory responses induced by exposure to the candidate is essential as a first assessment of mucosal safety. Several studies have defined immune soluble markers as biomarkers of inflammation in ex vivo tissue explant models [66,67,68]. Further to the results reported in the paper by Shah et al. [45] describing the lack of inflammatory proteomic responses to the AMPs by vaginal epithelial cells, we found, at the transcriptomic level, no increased mRNA levels associated with genes reported to be biomarkers of inflammation in cervicovaginal cells and tissues [52]. At the proteomic level, in endometrial cells, HEC-1-A, and tissues, ECEs, no significantly increased secretion of pro-inflammatory cytokines/chemokines was observed following exposure to the AMPs for 24 h (Figure 2). In tissues, MAT-006 and UCD-MAT-001, in general, induced a decrease in immune mediators such as IL-1β, IL-4, IL-10, IL-12, and TNF-α, likely associated with a quiescent tissue immune response. This anti-inflammatory/quiescent profile has been identified as part of their anti-microbial mechanism of action [69,70]. Furthermore, the anti-inflammatory proteomic responses in HEC-1-A cells and ECEs and associated functional pathways showing no activation of immune cells (Supplementary Figures S5–S8) are indicative of a positive mucosal safety profile for the topical use of the three human-milk-inspired AMPs.

AMPs were tested for their pre-exposure and post-exposure prophylactic antiviral activity. In this pilot study, two post-exposure time points were selected: 2 h, at which point viral entry in target cells has occurred, and 8 h, corresponding to a time point in the viral replication cycle post-entry and early reverse transcription, but prior to viral integration and the establishment of productive infection [71,72]. Furthermore, these two time points represent, from an end-user point of view, early and morning-after post-coital times for post-exposure prophylaxis, respectively. All three AMPs showed anti-HIV activity under co-exposure experimental conditions, while MAT-006 and UCD-MAT-001 also showed activity under post-exposure (2 h) conditions (Figure 4). Only MAT-006 showed antiviral activity under pre-exposure conditions. All three AMPs may have blocked the process of viral entry by binding to viral proteins or cellular receptors involved in the adhesion, binding, or fusion process [72]. Showing antiviral activity when co-incubated with cells and removed prior to viral exposure, M006 may have blocked cellular receptors or simply remained attached to the membrane until the virus was added to the culture medium. The three AMPs have been shown to permeabilize the membrane of bacteria associated with BV [45]; however, whether their antiviral activity is mediated by the disruption of the viral envelope, binding to viral proteins or to viral receptors on the surface of the target cell, or a combination of these mechanisms remains to be determined.

STI pathogens such as HIV-1 and HSV-2 are carried into the female genital tract by seminal plasma, which not only acts as a vehicle but modulates infection [73]. SP has been shown to reduce the efficacy of certain drugs [74,75] and to enhance HIV-1 [76,77,78,79,80] and HSV-2 infection [81]. In our experiments, the anti-HIV-1_BaL_ activity of the three AMPs was not significantly affected by SP; however, SP impaired the anti-HSV-2 inhibitory potency of UCD-MAT-002 (Figure 6). The interference of SP on polyanionic compounds with anti-HIV or HSV-2 activity has been ascribed to the blocking of these charges by semen polyamines which, at neutral pH, are positively charged [74,75]. The absence of observed interference with the AMPs’ anti-HIV activity points to AMP inhibitory mechanisms not mediated by electronegative charge binding. The effect of SP on the AMPs’ anti-HSV-2 activity was observed in an in vitro model mimicking multiple rounds of viral replication. This is different from the single-round infection of HIV-1 in TZM-bl cells and might point towards a different mechanism of interference against HSV-2 than against HIV-1, occurring at a later stage of the viral replication cycle not mimicked by the single-round TZM-bl cell model [82]. To better define the AMPs’ mechanisms of action, further studies would need to be performed, including, among others, competition experiments, more detailed time-of-addition assays, infection studies with cells expressing different viral receptors and co-receptors, and molecular docking studies [83,84,85].

This pilot study had some limitations. We characterized the antiviral activity of three human-milk-inspired AMPs which were selected based on their antibacterial activity against BV pathogens. Potentially, other peptides with lower antibacterial activity might have been more potent against HIV or HSV. We did not use scrambled AMPs as our control. We tested only HIV-1_BaL_ and one HSV-2 strain G isolate; HIV-1 transmitted founders and other HSV-2 isolates will be needed to fully characterize the degree of inhibition for these three AMPs. Additionally, although the models used in this study are routinely used for preclinical screening, they have limitations that need to be considered. In vitro models are often not appropriate to define sterilizing viral inhibition. Furthermore, the viral challenge titers used in vitro are likely different than those needed for in vivo transmission. TZM-bl cells are known to express CCR5 levels more than two logs higher than those found in physiological target cells [86], making this model more susceptible. In contrast, higher HSV-2 titers are required to infect HEC-1-A cells compared to the inoculum required for in vivo transmission [87]. The ex vivo ecto-cervical tissue explant model is a model that has been increasingly used for pre-clinical screening [72]; however, the cytokine/chemokine profile can be affected by the multiple steps of specimen processing, which could trigger inflammatory responses [71]. To minimize the potential masking of innate responses to AMPs by these method-induced responses, cytokine/chemokine profiles were measured no later than 72 h post-resection. The immune response profiles observed in ECEs warrant future studies evaluating the antiviral activity of the AMPs in more complex tissue systems such as ECEs prior to in vivo testing, which will help better define a safe and effective dose.

Considering the increasing emergence and spread of antibiotic and antiviral resistance [88,89,90,91,92,93], new antimicrobials with antibacterial and antiviral activity and a different mechanism of action from currently available products are highly desirable. The three human-milk-inspired AMPs evaluated in this study and by Shah et al. [45] are short peptides, relatively easy to synthesize, with no deleterious effect on healthy mucosal tissue and microbiota, which have shown natural broad antimicrobial activity preclinically. The results from this in vitro study represent the foundation for further preclinical evaluation in more complex models, which will inform the development of a selected AMP as a promising candidate for multi-purpose products to treat BV and prevent HIV-1 and HSV-2 acquisition topically in the female genital tract.

## Figures and Tables

**Table 1:** HSV-2 primer and probe sequences.

Primer	Sequence
HSV-2 Forward	5′-CGC ATC AAG ACC TCC TC-3′
HSV-2 Reverse	5′-AGC TTG CGG GCC TCG TT-3′
HSV-2 Probe	5′-CGC CCC AGC ATG TCG TTC ACG T-3
HSV-2 DNA template	cgccactgcg ggaggcgccc agcgccaacg cgtccgtgga gcgcatcaag accacctcct cgatcgagtt cgcccggctg cagtttacgt ataaccacat acagcgccac gtgaacgaca tgctggggcg catcgccgtc gcgtggtgcg agctgcagaa ccacgagctg actctctgga acgaggcccg caagctcaac cccaacgcca tcgcctccgc

**Table 2:** Anti-HIV-1_BaL_ activity of human-milk-inspired AMPs in TZM-bl cells.

Dosing Time		Inhibitory Concentrations (mg/mL) ^a^					
		MAT-006		UCD-MAT-001		UCD-MAT-002	
		IC_50_	IC_90_	IC_50_	IC_90_	IC_50_	IC_90_
Pre-exposure	1 h	7.79 (5.194)	NA				
	6 h	1.07 (0.515)	11.36 (5.855)	NA	NA	NA	NA
	24 h	0.69 (0.152)	6.43 (0.982)				
Post-exposure	2 h	0.82 (0.447)	5.95 (1.292)	2.62 (0.952)	10.50 (3.496)	7.72 (1.649)	17.42 (2.510)
	8 h	4.85 (1.487)	NA	2.30 (0.475)	NA	5.77 (1.047)	12.93 (2.354)
Co-exposure		1.29 (1.098)	4.57 (2.29)	0.76 (0.399)	4.10 (1.243)	1.47 (1.006)	6.59 (4.910)

^a^ Data are means (standard deviations) derived from at least 2 independent experiments performed in triplicate. NA: not applicable; values could not be calculated as 50% inhibition of infection was not achieved within the range of concentrations tested. Inhibitory concentrations >5 mg/mL were estimated from extrapolation of the sigmoidal dose–response curve.

**Table 3:** Anti-HSV-2 activity of human-milk-inspired AMPs in HEC-1-A cells.

AMP	Inhibitory Concentrations (mg/mL) ^a^	
	IC_50_	IC_90_
MAT-006	1.08 (0.736)	3.14 (1.825)
UCD-MAT-001	0.78 (0.296)	2.83 (0.206)
UCD-MAT-002	1.03 (0.568)	3.43 (0.456)

^a^ Data are means (standard deviations) derived from at least 3 independent experiments performed in triplicate.

## Data Availability

Data are available upon request to the corresponding author.

## Supplementary Materials

The following supporting information can be downloaded at: https://www.mdpi.com/article/10.3390/pharmaceutics18080938/s1, Figure S1: HEC-1-A-cell responses to milk AMPs following 24 h exposure; Figure S2: Ectocervical responses to milk AMPs following 24 h ex vivo exposure; Figure S3: HEC-1-A cell responses to milk AMPs following 48 h exposure; Figure S4: Ectocervical responses to milk AMPs following 48 h ex vivo exposure; Figure S5: Functional pathways associated with exposure of HEC-1-A cells to human-milk-inspired AMPs; Figure S6: Canonical pathways significantly enriched in culture supernatants of HEC-1-A cell exposed to human-milk-inspired AMPs; Figure S7: Functional pathways associated with exposure of ecto-cervical tissue explants to human-milk-inspired AMPs; Figure S8: Canonical pathways significantly enriched in culture supernatants of ecto-cervical tissue explants exposed to human-milk-inspired AMPs.

## List of Abbreviations

AMPs: Antimicrobial peptides; APOBEC: Apolipoprotein B mRNA editing catalytic polypeptide-like; ATCC: American Type Culture Collection; BV: Bacterial vaginosis; CCL20: C-C chemokine ligand 20; CCR5 C-C chemokine receptor type 5; CD4: cluster of differentiation 4; CLDN1: Claudin-1; cRPMI: complete Roswell Park Memorial Institute; CXCL2: C-X-C chemokine ligand 2; DMEM: Dulbecco’s Modified Eagle Medium; ECEs: Ectocervical explants; EGF: Epidermal growth factor; FBS: Fetal bovine serum; FDA: Food and Drug Administration; FGT: Female genital tract; HEC: Hydroxyethyl cellulose; HIV-1_BaL_: Human immunodeficiency virus type 1 BaL; HSV-2: Herpes Simplex virus type 2; IC_50_: 50 percent inhibitory concentration; IC_90_: 90 percent inhibitory concentration; IFN-γ: Interferon-γ; IL: Interleukin; IMQ: Imiquimod; MSD: Meso Scale Discovery; MOI: Multiplicity of infection; MTS: 3-(4,5-dimethylthiazol-2-yl)-5-(3-carboxymethoxyphenyl)-2-(4-sulfophenyl)-2H-tetrazolium; MTT: 3-(4,5-dimethylthiazol-2-yl)-2,5-diphenyltetrazolium bromide; pH: potential of hydrogen; pfu: Plaque forming unit; qRT-PCR: Quantitative real-time polymerase chain reaction; RLU: Relative light units; r.p.m: Revolutions per minute; SP: Seminal plasma; STI: Sexually transmitted infection; T20: Enfuvirtide; TCID_50_: Tissue Culture Infectious Dose 50%; TLR: Toll-like receptor; TFV: Tenofovir; TNF-α: Tumor necrosis factor-α; UNAIDS: United Nations Programme on HIV/AIDS.
