# Supplementary material for: Antiviral Activity and Mucosal Safety of Novel Human-Milk-Inspired Antimicrobial Peptides
Source: Pharmaceutics. 2026 Jul 30;18(8):938. doi: 10.3390/pharmaceutics18080938 (PMC13516257; doi:10.3390/pharmaceutics18080938)
Supplement: Supplementary file 1 [file pharmaceutics-18-00938-s001.zip › pharmaceutics-4382906-supplementary.pdf]

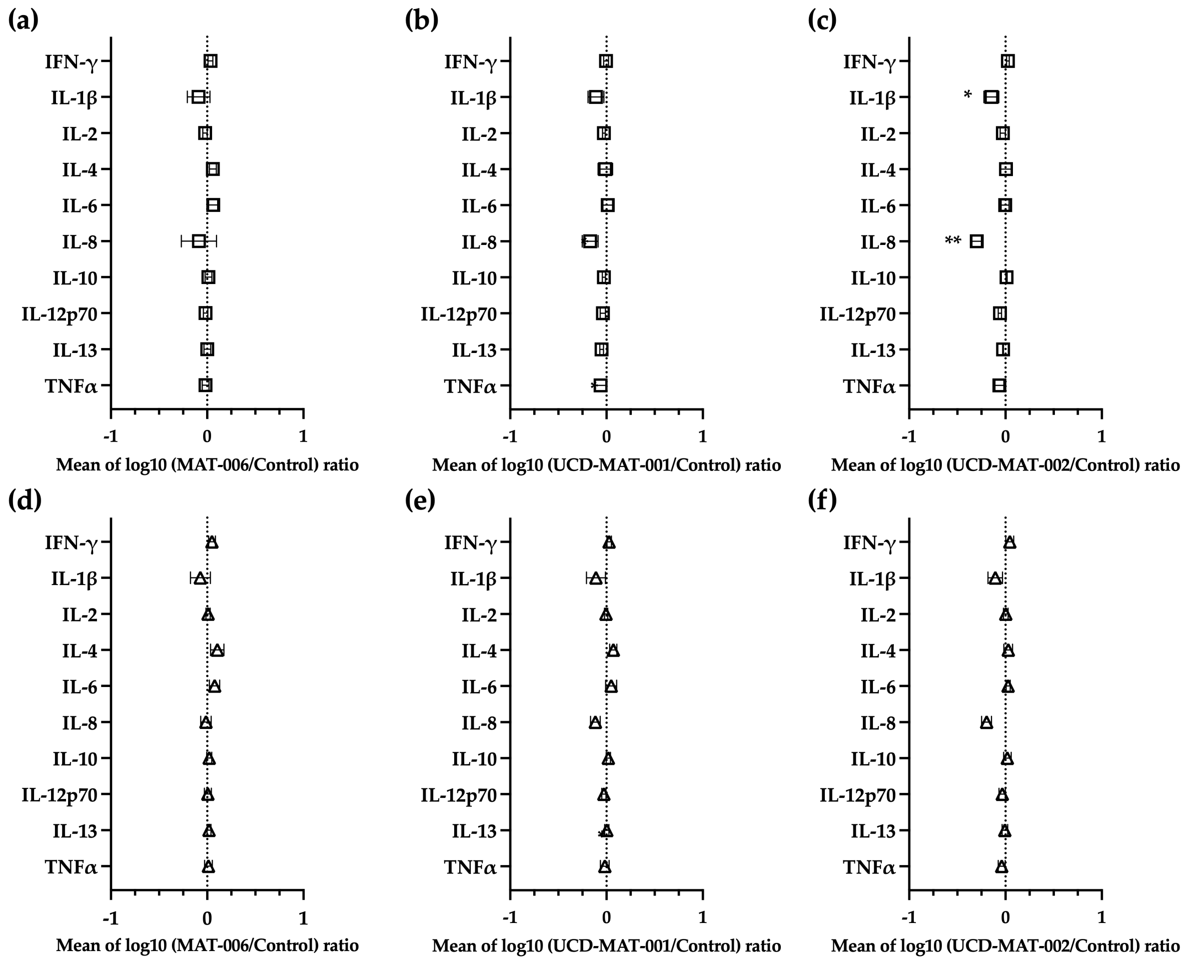

Figure S1. HEC-1-A-cell responses to milk AMPs following 24 h exposure. Endometrial HEC-1-A cells were treated with MAT-006 (**a**, **d**), UCD-MAT-001 (**b**, **e**), or UCD-MAT-002 (**c**, **f**) at 5 mg/mL (**a**, **b**, **c**) or 1 mg/mL (**d**, **e**, **f**) for 24 h. Data shown are mean log concentrations of treatments vs. control ratios from at least two independent experiments performed in duplicate. Statistical analyses between treatment and control untreated samples were conducted using ordinary one-way ANOVA with Tukey's multiple comparisons tests. *$p<0.05$, **$p<0.01$, ***$p<0.001$, ****$p<0.0001$.

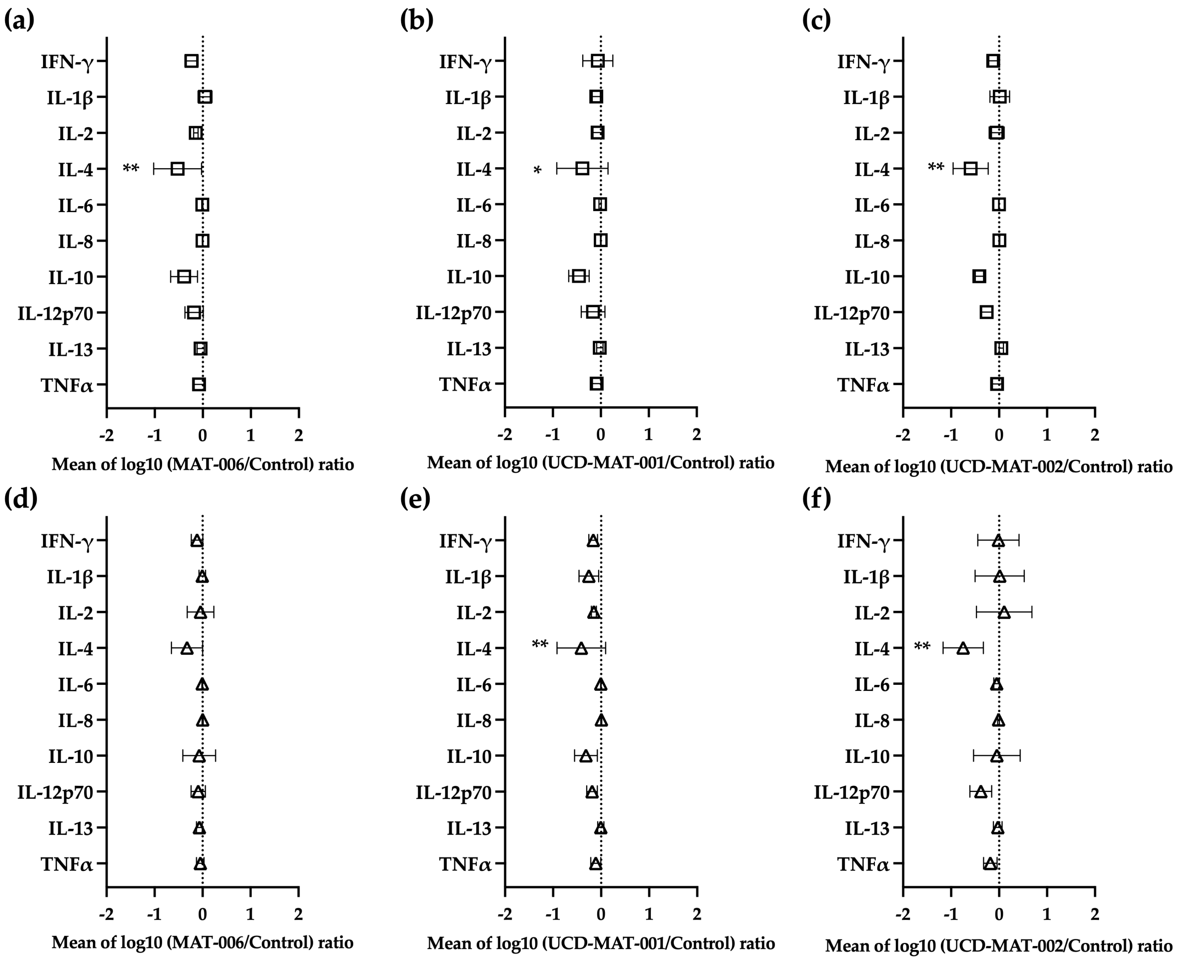

Figure S2. Ectocervical responses to milk AMPs following 24 h *ex vivo* exposure. Ectocervical tissue explants were treated with MAT-006 (**a**, **d**), UCD-MAT-001 (**b**, **e**), or UCD-MAT-002 (**c**, **f**) at 5 mg/mL (**a**, **b**, **c**) or 1 mg/mL (**d**, **e**, **f**) for 24 h. Data shown are mean log concentrations of treatments vs. control ratios from at least two independent experiments performed in duplicate. Statistical analyses between treatment and control untreated samples were conducted using ordinary one-way ANOVA with Tukey's multiple comparisons tests. *$p<0.05$, **$p<0.01$, ***$p<0.001$, ****$p<0.0001$.

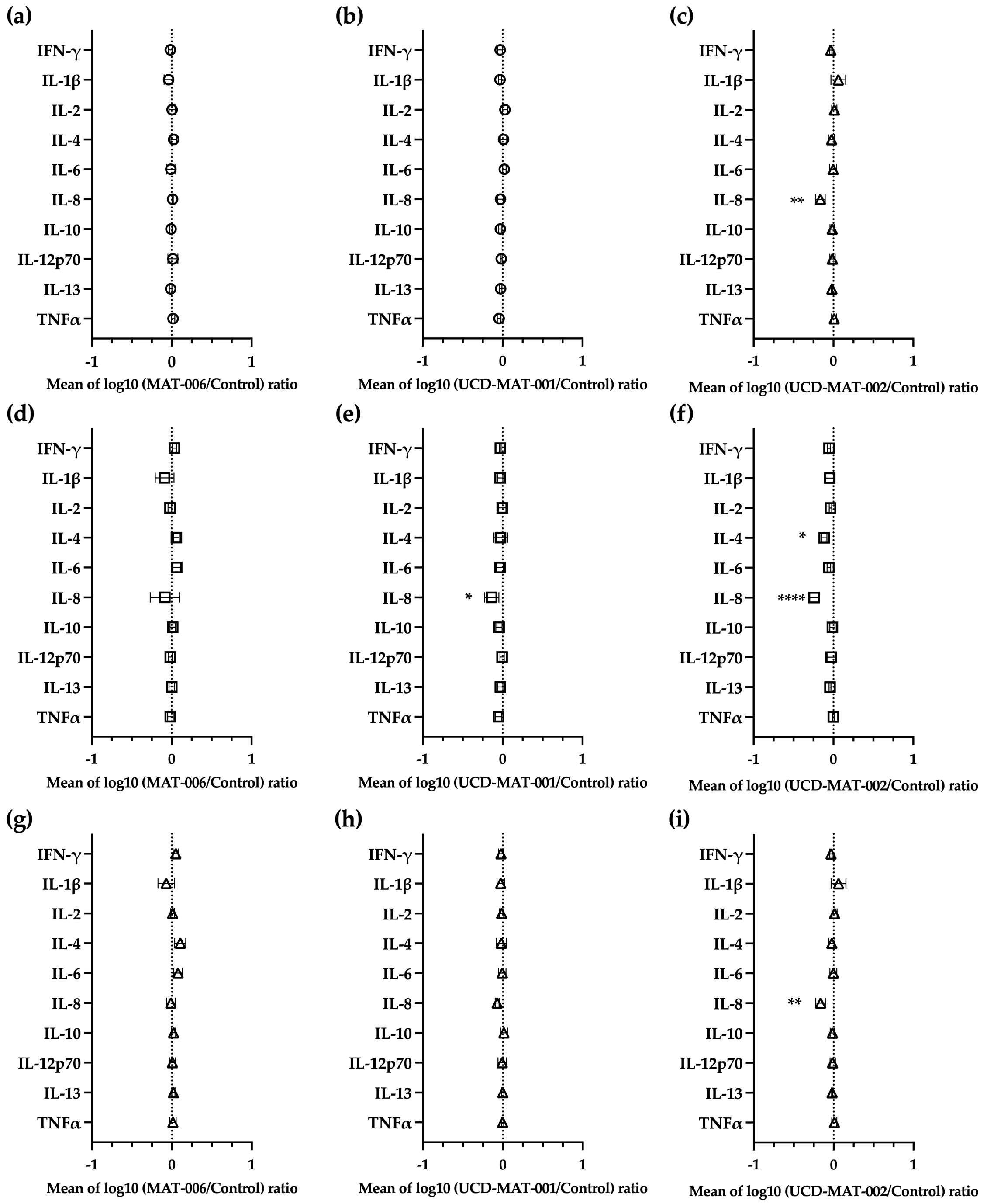

Figure S3. HEC-1-A cell responses to milk AMPs following 48 h exposure. Endometrial HEC-1-A cells were treated with MAT-006 (**a**, **d**, **g**), UCD-MAT-001 (**b**, **e**, **h**), or UCD-MAT-002 (**c**, **f**, **i**) at 10 mg/mL (**a**, **b**, **c**), 5 mg/mL (**d**, **e**, **f**) or 1 mg/mL (**g**, **h**, **i**) for 48 h. Data shown are mean log concentrations of treatments vs. control ratios from at least two independent experiments performed in duplicate. Statistical analyses between treatment and control untreated samples were conducted using ordinary one-way ANOVA with Tukey's multiple comparisons tests. *$p<0.05$, **$p<0.01$, ***$p<0.001$, ****$p<0.0001$.

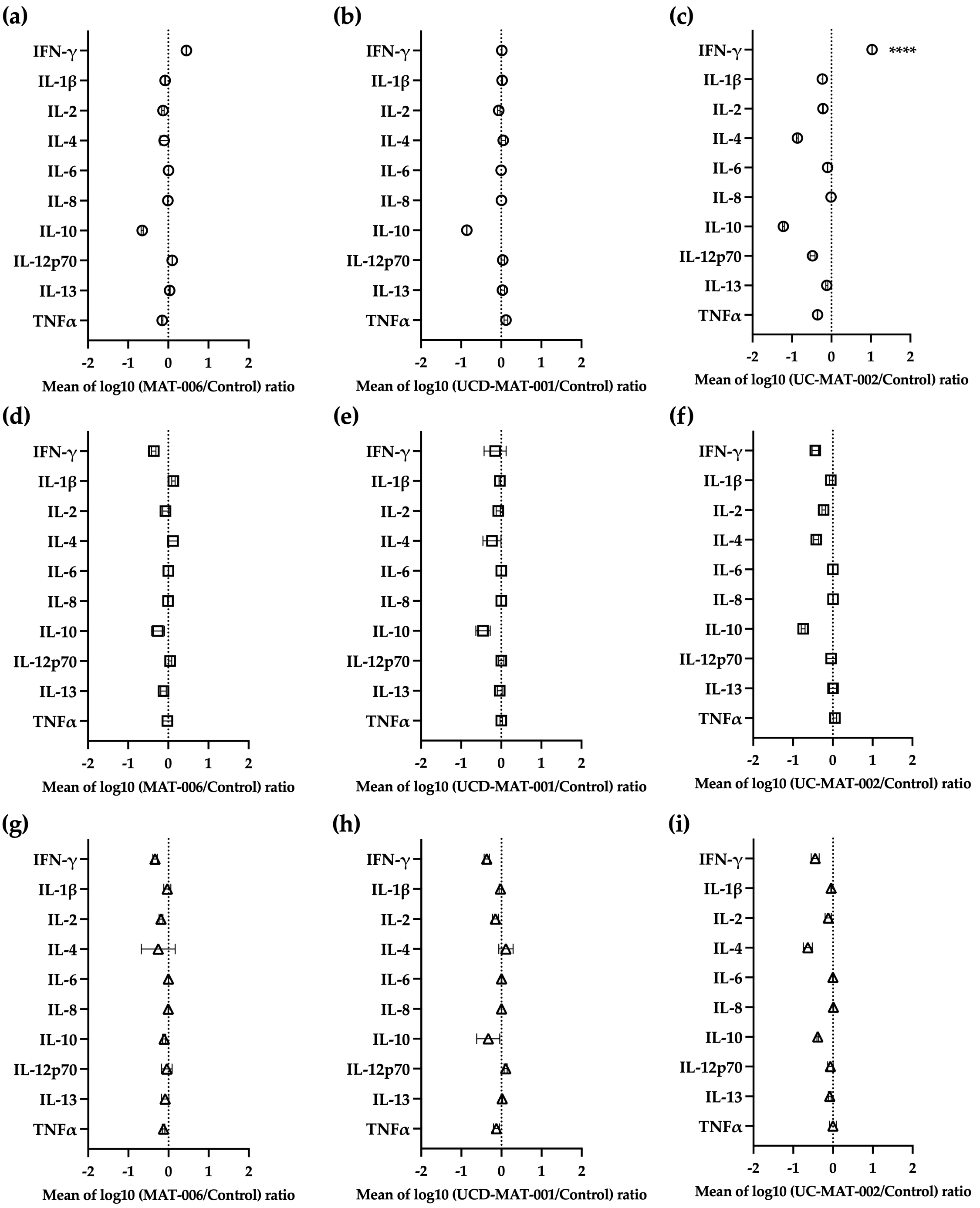

Figure S4. Ectocervical responses to milk AMPs following 48 h *ex vivo* exposure. Ectocervical tissue explants were treated with MAT-006 (**a**, **d**, **g**), UCD-MAT-001 (**b**, **e**, **h**), or UCD-MAT-002 (**c**, **f**, **i**) at 10 mg/mL (**a**, **b**, **c**), 5 mg/mL (**d**, **e**, **f**) or 1 mg/mL (**g**, **h**, **i**) for 48 h. Data shown are mean log concentrations of treatments vs. control ratios from at least two independent experiments performed in duplicate. Statistical analyses between treatment and control untreated samples were conducted using ordinary one-way ANOVA with Tukey's multiple comparisons tests. *p<0.05, **p<0.01, ***p<0.001, ****p<0.0001.

(a)

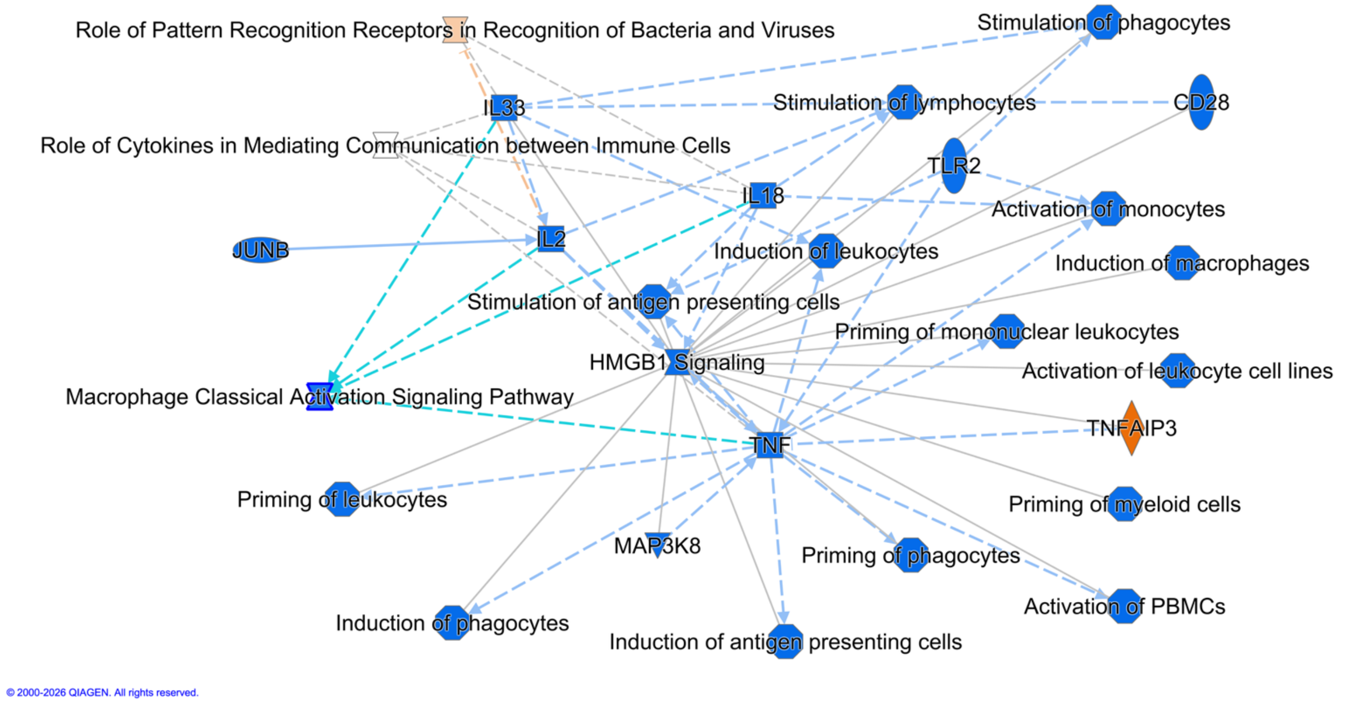

(b)

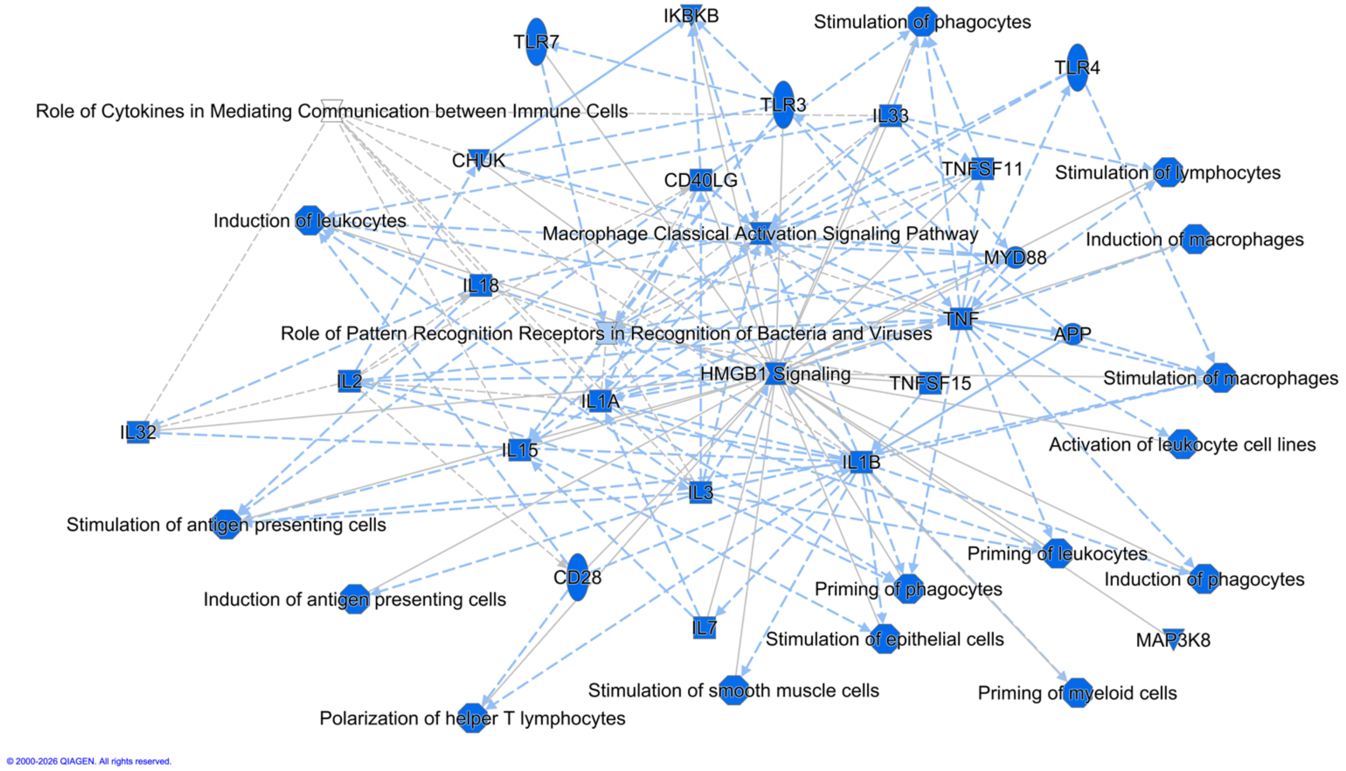

Figure S5. Functional pathways associated with exposure of HEC-1-A cells to human-milk-inspired AMPs. Interaction maps of the functional pathways associated with the cytokine/chemokine nodes modulated in HEC-1-A cells after 24 h of incubation with 10 µg/mL of (**a**) UCD-MAT-001, or (**b**) UCD-MAT-002. Colors: blue: downregulated; orange: upregulated. Symbols: oval: transmembrane receptor; square: cytokine/chemokine; diamond: enzyme; inverted triangle: kinase; hourglass: canonical pathway; octagon: function. Lines: solid: direct interaction; dashed: indirect interaction; doted: inferred correlation from Ingenuity Pathway Analysis machine-based learning.

(a)

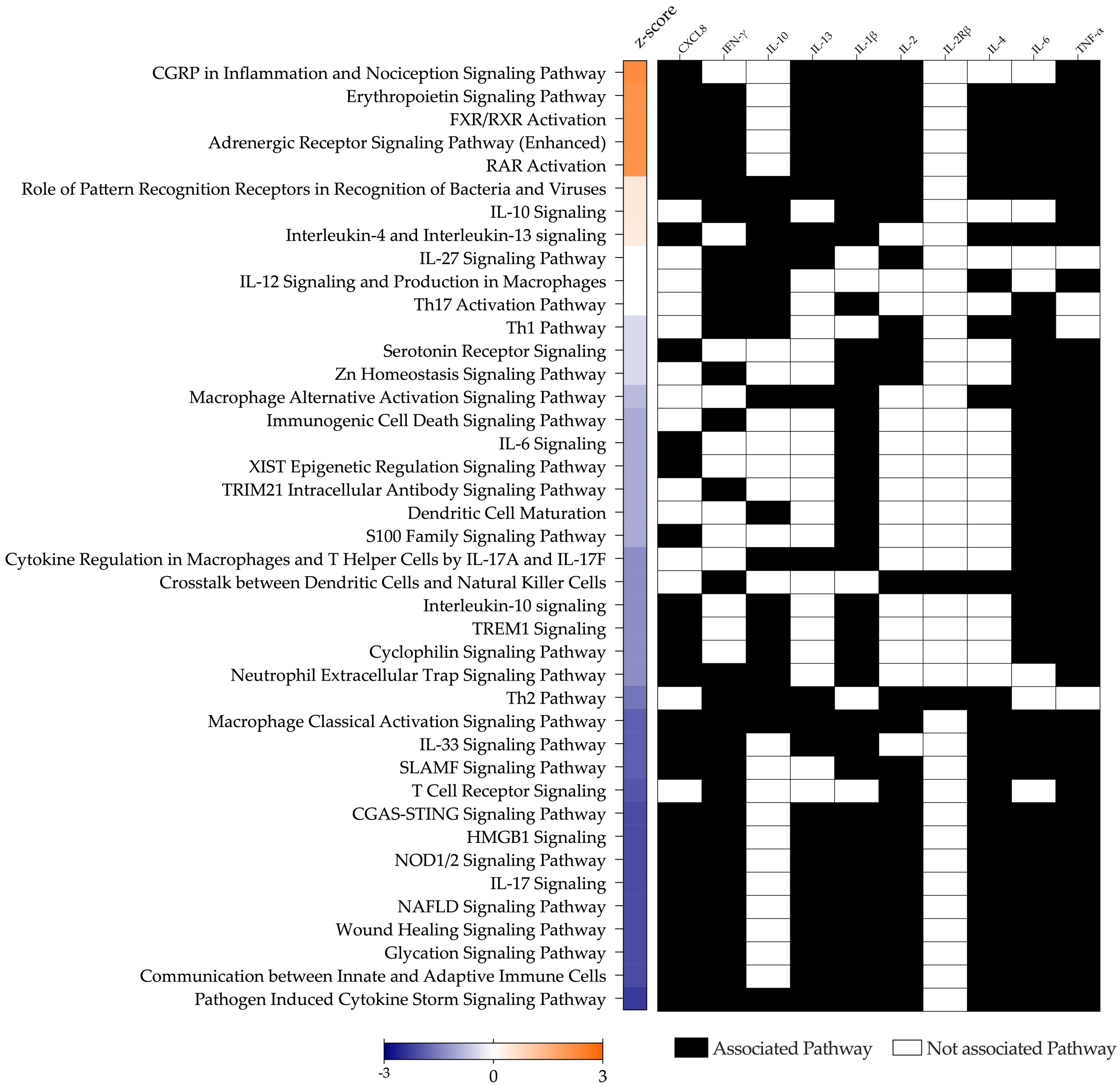
Z-score
CXCL8
IFN-γ
IL-10
IL-13
IL-1β
IL-2
IL-2Rβ
IL-4
IL-6
TNF-α
CGRP in Inflammation and Nociception Signaling Pathway
Erythropoietin Signaling Pathway
FXR/RXR Activation
Adrenergic Receptor Signaling Pathway (Enhanced)
RAR Activation
Role of Pattern Recognition Receptors in Recognition of Bacteria and Viruses
IL-10 Signaling
Interleukin-4 and Interleukin-13 signaling
IL-27 Signaling Pathway
IL-12 Signaling and Production in Macrophages
Th17 Activation Pathway
Th1 Pathway
Serotonin Receptor Signaling
Zn Homeostasis Signaling Pathway
Macrophage Alternative Activation Signaling Pathway
Immunogenic Cell Death Signaling Pathway
IL-6 Signaling
XIST Epigenetic Regulation Signaling Pathway
TRIM21 Intracellular Antibody Signaling Pathway
Dendritic Cell Maturation
S100 Family Signaling Pathway
Cytokine Regulation in Macrophages and T Helper Cells by IL-17A and IL-17F
Crosstalk between Dendritic Cells and Natural Killer Cells
Interleukin-10 signaling
TREM1 Signaling
Cyclophilin Signaling Pathway
Neutrophil Extracellular Trap Signaling Pathway
Th2 Pathway
Macrophage Classical Activation Signaling Pathway
IL-33 Signaling Pathway
SLAMF Signaling Pathway
T Cell Receptor Signaling
CGAS-STING Signaling Pathway
HMGB1 Signaling
NOD1/2 Signaling Pathway
IL-17 Signaling
NAFLD Signaling Pathway
Wound Healing Signaling Pathway
Glycation Signaling Pathway
Communication between Innate and Adaptive Immune Cells
Pathogen Induced Cytokine Storm Signaling Pathway
-3
0
3
Associated Pathway
Not associated Pathway

(b)

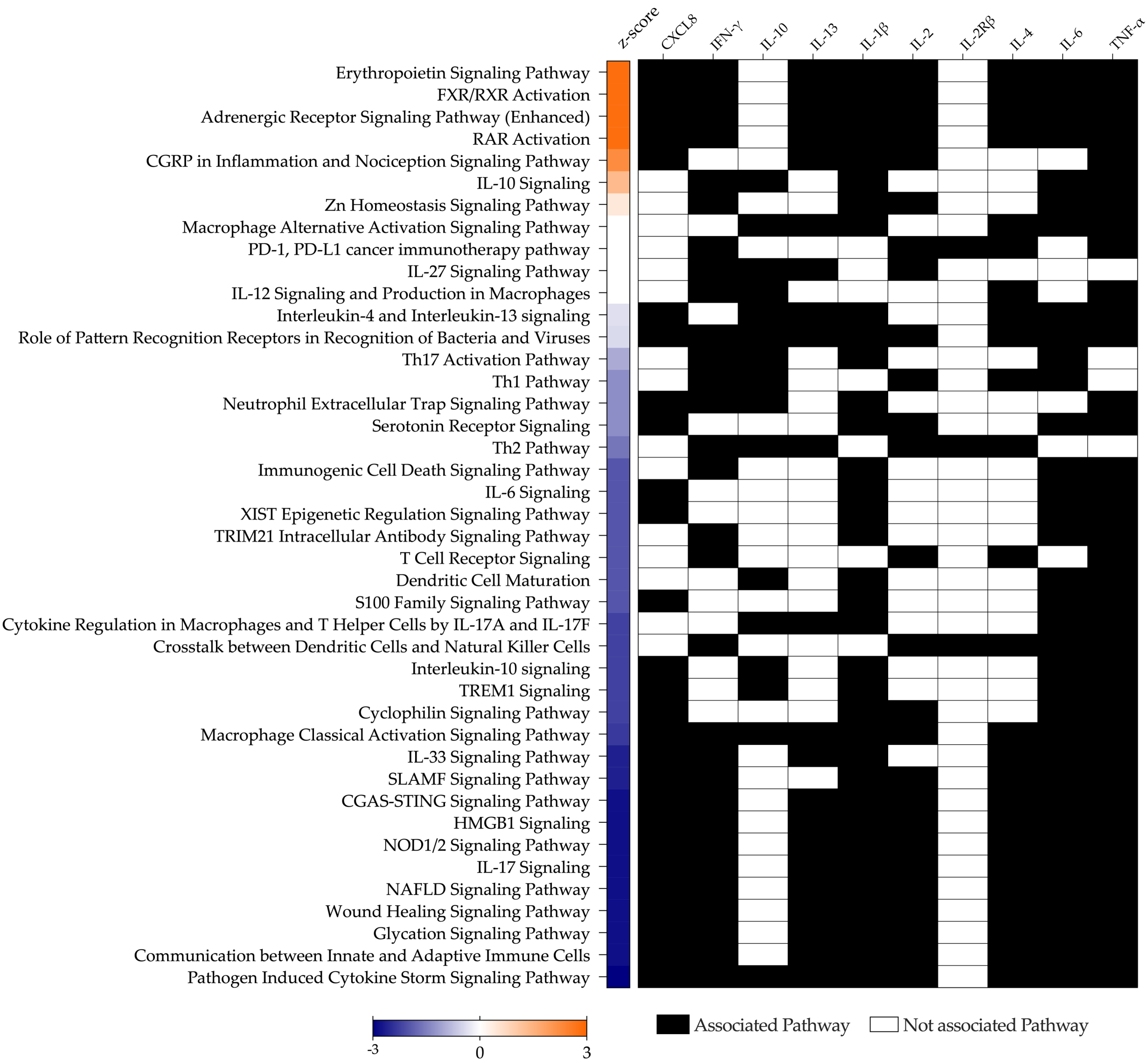

Figure S6. Canonical pathways significantly enriched in culture supernatants of HEC-1-A cell exposed to human-milk-inspired AMPs. The activation z-scores, after 24 h incubation of HEC-1-A cells with 10 µg/mL of (**a**) UCD-MAT-001, or (**b**) UCD-MAT-002 compared to untreated cells, determined by directionality (overabundance in orange and underabundance in blue) and number of cytokine/chemokine are represented by a heat map. The proteins associated to each pathway are shown in black cells. The pathways included had a minimum of two analytes associated and a $p<0.05$ (right-tailed Fisher exact test).

(a)

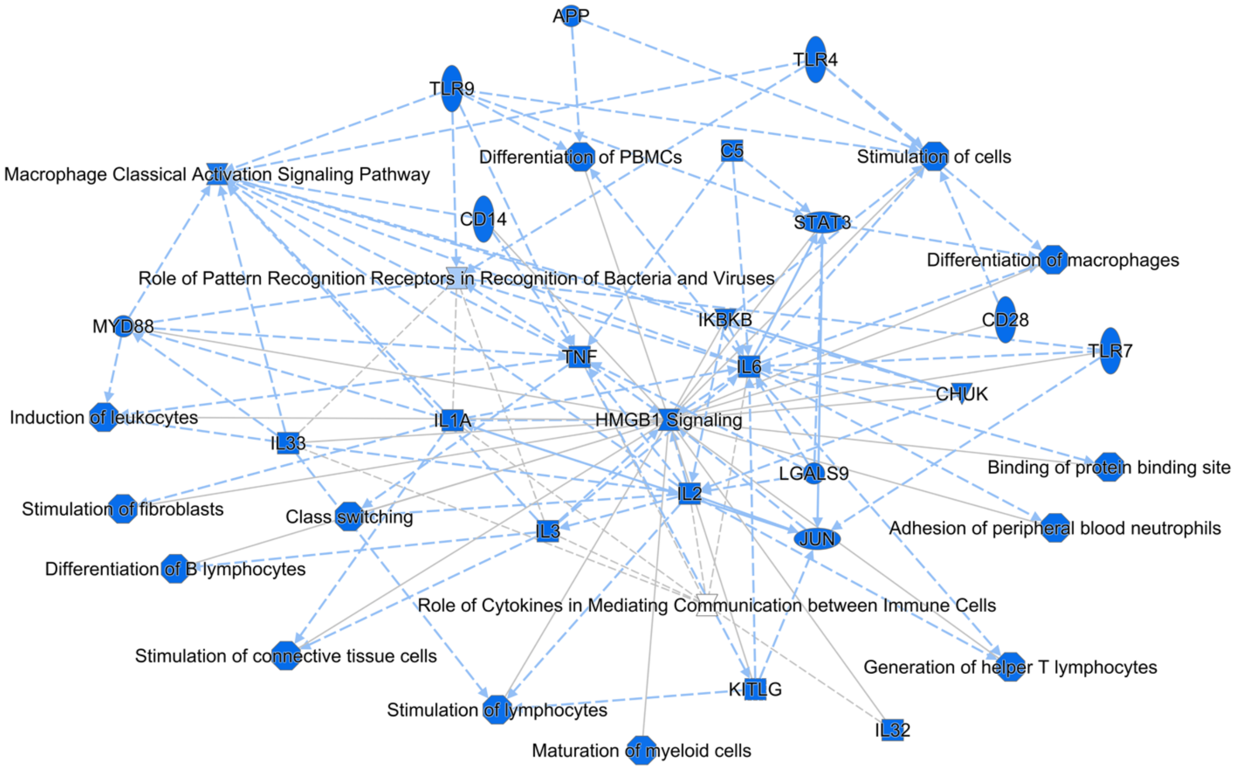

© 2000-2026 QIAGEN. All rights reserved.

(b)

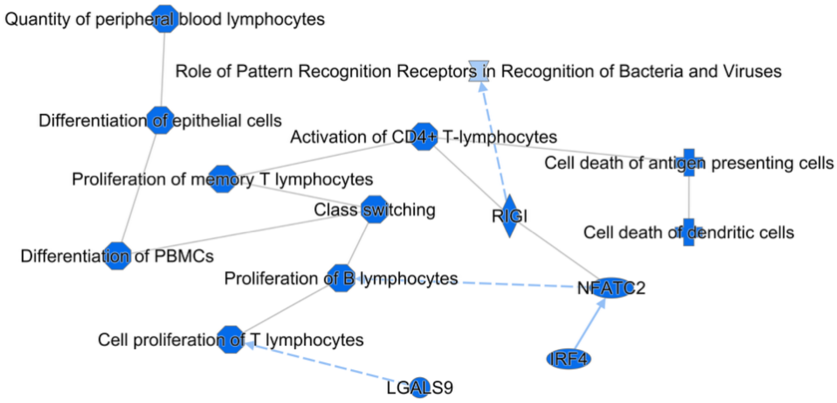

© 2000-2026 QIAGEN. All rights reserved.

(c)

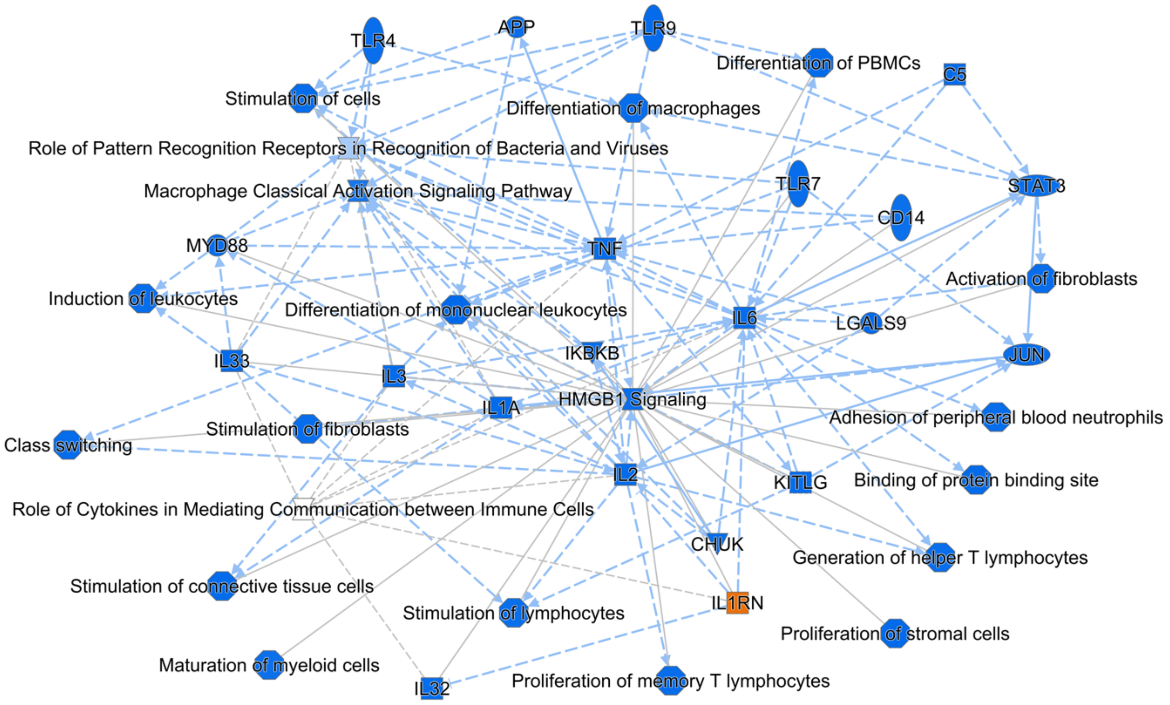

© 2000-2026 QIAGEN. All rights reserved.

Figure S7. Functional pathways associated with exposure of ecto-cervical tissue explants to human-milk-inspired AMPs. Interaction maps of the functional pathways associated with the cytokine/chemokine nodes modulated in ecto-cervical explants after 24 h of incubation with 10 μg/mL of (**a**) MAT-006, (**b**) UCD-MAT-001, or (**c**) UCD-MAT-002. Colors: blue: downregulated; orange: upregulated. Symbols: oval: transmembrane receptor; square: cytokine/chemokine; diamond: enzyme; inverted triangle: kinase; hourglass: canonical pathway; octagon: function; cross: disease. Lines: solid: direct interaction; dashed: indirect interaction; doted: inferred correlation from Ingenuity Pathway Analysis machine-based learning.

(a)

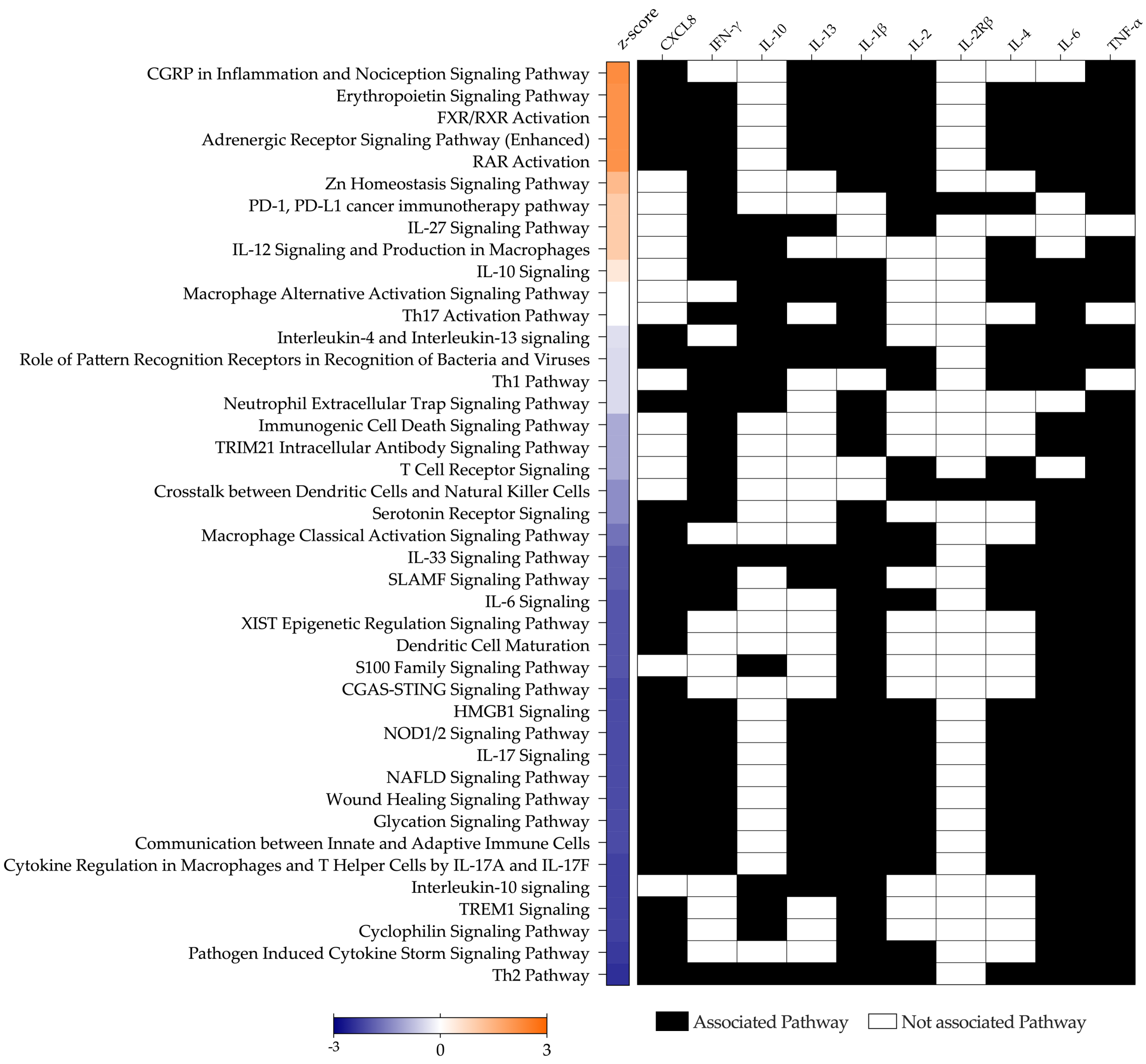
z-score
CXCL8
IFN-γ
IL-10
IL-13
IL-1β
IL-2
IL-2Rβ
IL-4
IL-6
TNF-α
CGRP in Inflammation and Nociception Signaling Pathway
Erythropoietin Signaling Pathway
FXR/RXR Activation
Adrenergic Receptor Signaling Pathway (Enhanced)
RAR Activation
Zn Homeostasis Signaling Pathway
PD-1, PD-L1 cancer immunotherapy pathway
IL-27 Signaling Pathway
IL-12 Signaling and Production in Macrophages
IL-10 Signaling
Macrophage Alternative Activation Signaling Pathway
Th17 Activation Pathway
Interleukin-4 and Interleukin-13 signaling
Role of Pattern Recognition Receptors in Recognition of Bacteria and Viruses
Th1 Pathway
Neutrophil Extracellular Trap Signaling Pathway
Immunogenic Cell Death Signaling Pathway
TRIM21 Intracellular Antibody Signaling Pathway
T Cell Receptor Signaling
Crosstalk between Dendritic Cells and Natural Killer Cells
Serotonin Receptor Signaling
Macrophage Classical Activation Signaling Pathway
IL-33 Signaling Pathway
SLAMF Signaling Pathway
IL-6 Signaling
XIST Epigenetic Regulation Signaling Pathway
Dendritic Cell Maturation
S100 Family Signaling Pathway
CGAS-STING Signaling Pathway
HMGB1 Signaling
NOD1/2 Signaling Pathway
IL-17 Signaling
NAFLD Signaling Pathway
Wound Healing Signaling Pathway
Glycation Signaling Pathway
Communication between Innate and Adaptive Immune Cells
Cytokine Regulation in Macrophages and T Helper Cells by IL-17A and IL-17F
Interleukin-10 signaling
TREM1 Signaling
Cyclophilin Signaling Pathway
Pathogen Induced Cytokine Storm Signaling Pathway
Th2 Pathway
-3
0
3
Associated Pathway
Not associated Pathway

(b)

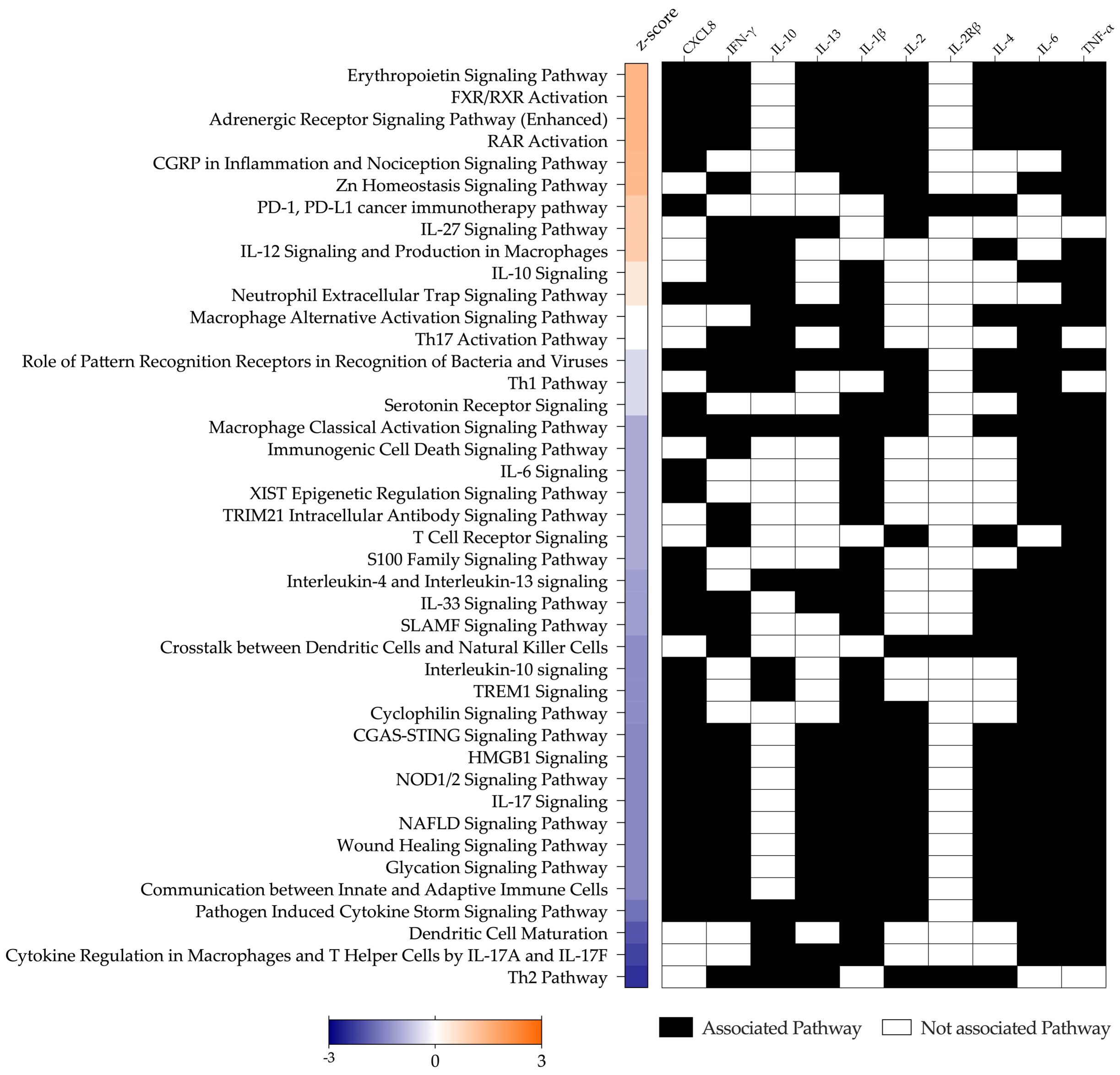

z-score
CXCL8
IFN-γ
IL-10
IL-13
IL-1β
IL-2
IL-2Rβ
IL-4
IL-6
TNF-α
Erythropoietin Signaling Pathway
FXR/RXR Activation
Adrenergic Receptor Signaling Pathway (Enhanced)
RAR Activation
CGRP in Inflammation and Nociception Signaling Pathway
Zn Homeostasis Signaling Pathway
PD-1, PD-L1 cancer immunotherapy pathway
IL-27 Signaling Pathway
IL-12 Signaling and Production in Macrophages
IL-10 Signaling
Neutrophil Extracellular Trap Signaling Pathway
Macrophage Alternative Activation Signaling Pathway
Th17 Activation Pathway
Role of Pattern Recognition Receptors in Recognition of Bacteria and Viruses
Th1 Pathway
Serotonin Receptor Signaling
Macrophage Classical Activation Signaling Pathway
Immunogenic Cell Death Signaling Pathway
IL-6 Signaling
XIST Epigenetic Regulation Signaling Pathway
TRIM21 Intracellular Antibody Signaling Pathway
T Cell Receptor Signaling
S100 Family Signaling Pathway
Interleukin-4 and Interleukin-13 signaling
IL-33 Signaling Pathway
SLAMF Signaling Pathway
Crosstalk between Dendritic Cells and Natural Killer Cells
Interleukin-10 signaling
TREM1 Signaling
Cyclophilin Signaling Pathway
CGAS-STING Signaling Pathway
HMGB1 Signaling
NOD1/2 Signaling Pathway
IL-17 Signaling
NAFLD Signaling Pathway
Wound Healing Signaling Pathway
Glycation Signaling Pathway
Communication between Innate and Adaptive Immune Cells
Pathogen Induced Cytokine Storm Signaling Pathway
Dendritic Cell Maturation
Cytokine Regulation in Macrophages and T Helper Cells by IL-17A and IL-17F
Th2 Pathway
-3
0
3
Associated Pathway
Not associated Pathway

(c)

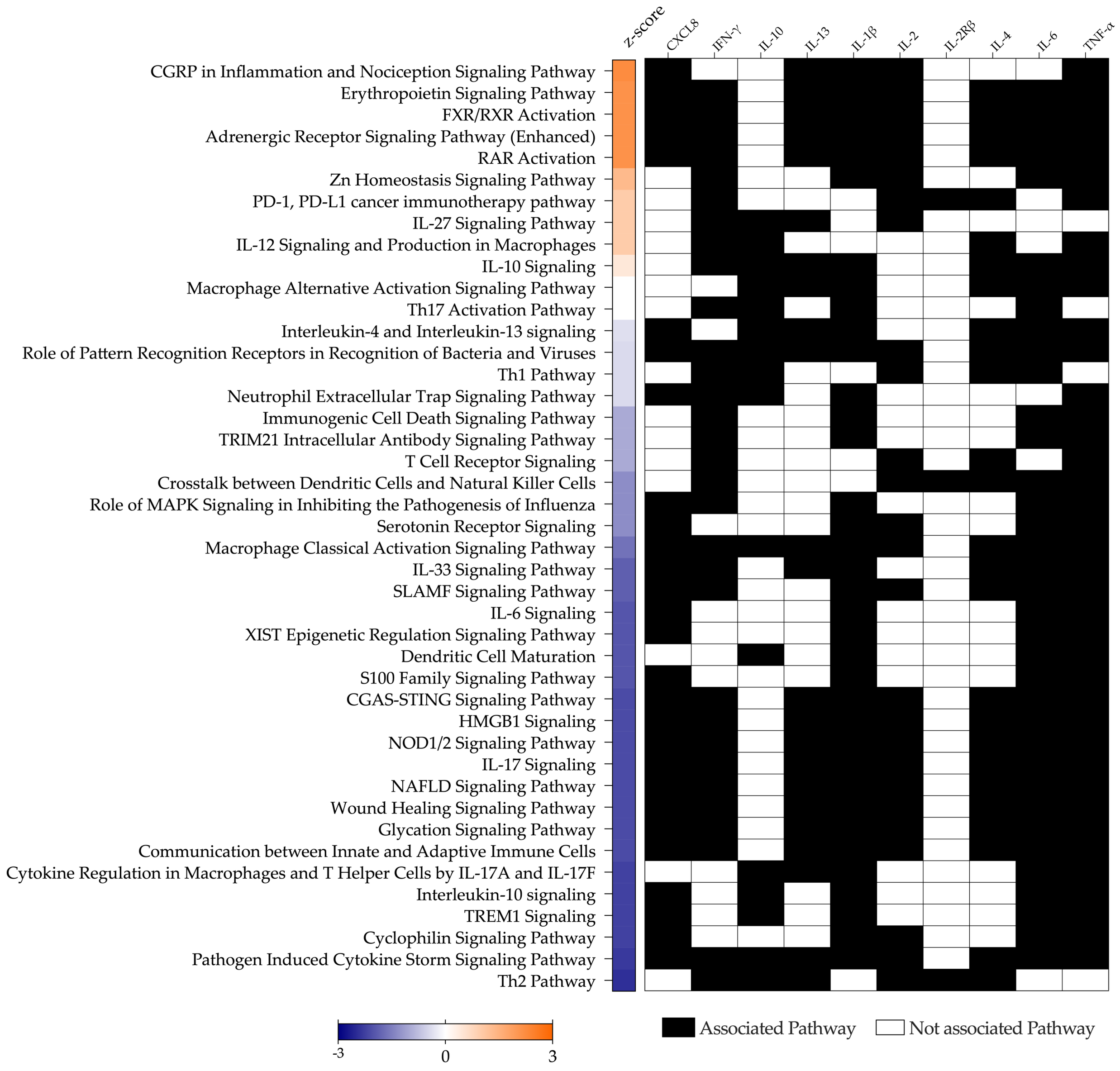

Figure S8. Canonical pathways significantly enriched in culture supernatants of ecto-cervical tissue explants exposed to human-milk-inspired AMPs. The activation z-scores, after 24 h incubation of explants with 10 µg/mL of (**a**) MAT-006, (**b**) UCD-MAT-001, or (**c**) UCD-MAT-002 compared to untreated explants, determined by directionality (overabundance in orange and underabundance in blue) and number of cytokine/chemokine are represented by a heat map. The proteins associated to each pathway are shown in black cells. The pathways included had a minimum of two analytes associated and a $p<0.05$ (right-tailed Fisher exact test).
